# Photocatalytic Overall Water Splitting Under Visible Light Enabled by a Particulate Conjugated Polymer Loaded with Palladium and Iridium**

**DOI:** 10.1002/anie.202201299

**Published:** 2022-04-26

**Authors:** Yang Bai, Chao Li, Lunjie Liu, Yuichi Yamaguchi, Mounib Bahri, Haofan Yang, Adrian Gardner, Martijn A. Zwijnenburg, Nigel D. Browning, Alexander J. Cowan, Akihiko Kudo, Andrew I. Cooper, Reiner Sebastian Sprick

**Affiliations:** ^1^ Materials Innovation Factory & Department of Chemistry University of Liverpool Liverpool L7 3NY UK; ^2^ Institute of Materials Research and Engineering Agency for Science Technology and Research Singapore 138634 Singapore; ^3^ Stephenson Institute for Renewable Energy University of Liverpool Liverpool L69 7ZF UK; ^4^ Department of Applied Chemistry Tokyo University of Science Tokyo 162-8601 Japan; ^5^ Albert Crewe Centre for Electron Microscopy University of Liverpool Liverpool L69 3GL UK; ^6^ Department of Chemistry University College London London WC1H 0AJ UK; ^7^ Department of Pure and Applied Chemistry University of Strathclyde Glasgow G1 1XL UK

**Keywords:** Co-Catalysts, Organic Photocatalysts, Overall Water Splitting

## Abstract

Polymer photocatalysts have received growing attention in recent years for photocatalytic hydrogen production from water. Most studies report hydrogen production with sacrificial electron donors, which is unsuitable for large‐scale hydrogen energy production. Here we show that the palladium/iridium oxide‐loaded homopolymer of dibenzo[*b*,*d*]thiophene sulfone (P10) facilitates overall water splitting to produce stoichiometric amounts of H_2_ and O_2_ for an extended period (>60 hours) after the system stabilized. These results demonstrate that conjugated polymers can act as single component photocatalytic systems for overall water splitting when loaded with suitable co‐catalysts, albeit currently with low activities. Transient spectroscopy shows that the IrO_2_ co‐catalyst plays an important role in the generation of the charge separated state required for water splitting, with evidence for fast hole transfer to the co‐catalyst.

## Graphical abstract

Poly(dibenzo[*b*,*d*]thiophene sulfone) loaded with Pd/IrO_2_ facilitates stoichiometric overall water splitting for an extended period after the system stabilized, albeit currently with low activities. The study demonstrates the importance of co‐catalysts for polymer photoatalysts and results were rationalized by transient absorption spectroscopy.

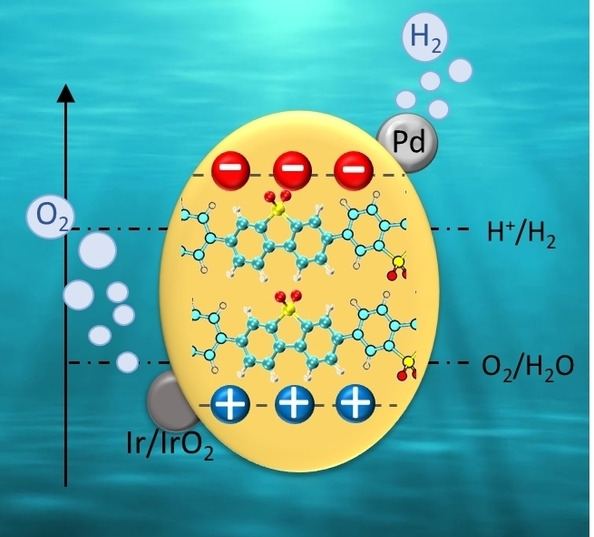

## Introduction

Photocatalytic water splitting using semiconductor photocatalysts has been studied extensively for the past few decades.[^1^, ^2^, ^3^, ^4^, ^5^, ^6^] Photoelectrochemical[^3^, ^6^, ^7^] and direct photocatalysis[^1^, ^2^, ^3^, ^4^, ^5^, ^6^, ^7^, ^8^, ^9^] using particulate catalyst suspensions both have been explored. In principle, overall water splitting using photocatalyst suspensions is the simplest approach in technological terms, providing that the two gases can be separated economically. Photochemical systems could be amenable to large‐scale deployment, potentially to a level that is competitive with fossil‐fuel‐derived hydrogen.[^2^, ^8^]

Most particulate semiconductor photocatalysts reported to date are inorganic materials,[^1^, ^2^, ^3^, ^4^, ^5^, ^6^, ^7^, ^8^, ^9^, ^10^, ^11^, ^12^, ^13^, ^14^, ^15^] but one well known challenge is to design materials that function in the visible part of the spectrum, as well as the UV. In the last decade, organic materials have shown promise due to their tunability (e.g., in terms of light absorption), and their potential to be produced inexpensively on large scale.^[10]^ Although organic photocatalysts were investigated widely after the first report of carbon nitride in 2009,^[16]^ most studies have been confined to sacrificial half‐reactions that produce either hydrogen or oxygen, not both.[^17^, ^18^, ^19^, ^20^, ^21^] Few organic photocatalysts have been reported for overall water splitting. Carbon nitride materials have been coupled with metal oxides to facilitate overall water splitting in so called Z‐schemes, whereby hydrogen evolution occurs on the organic photocatalyst while oxygen evolution occurs on the metal oxide. Both photocatalysts are excited and charges are transferred between the catalysts using redox mediators.[^22^, ^23^, ^24^, ^25^] Similarly, we reported a Z‐scheme for overall water splitting using a homopolymer of dibenzo[*b*,*d*]thiophene sulfone (P10) as the hydrogen evolution catalyst, coupled with BiVO_4_ acting as the oxygen evolution catalyst using a Fe^2+^/Fe^3+^ redox mediator system.^[26]^ Overall water splitting occurred, but the solar‐to‐hydrogen efficiency was very low.

The use of redox mediators in Z‐schemes can result in limitations arising from the kinetics of diffusion to and from the surface of the photocatalysts, surface interactions, and charge transfer between the mediator and the photocatalyst. The redox mediator can also result in potential sacrificial light absorption and the kinetics of both half reactions can be difficult to balance to facilitate overall water splitting with high efficiencies. Systems that use conductive layers are an alternative, but these also come with challenges in their fabrication.^[27]^

**Figure 1:**
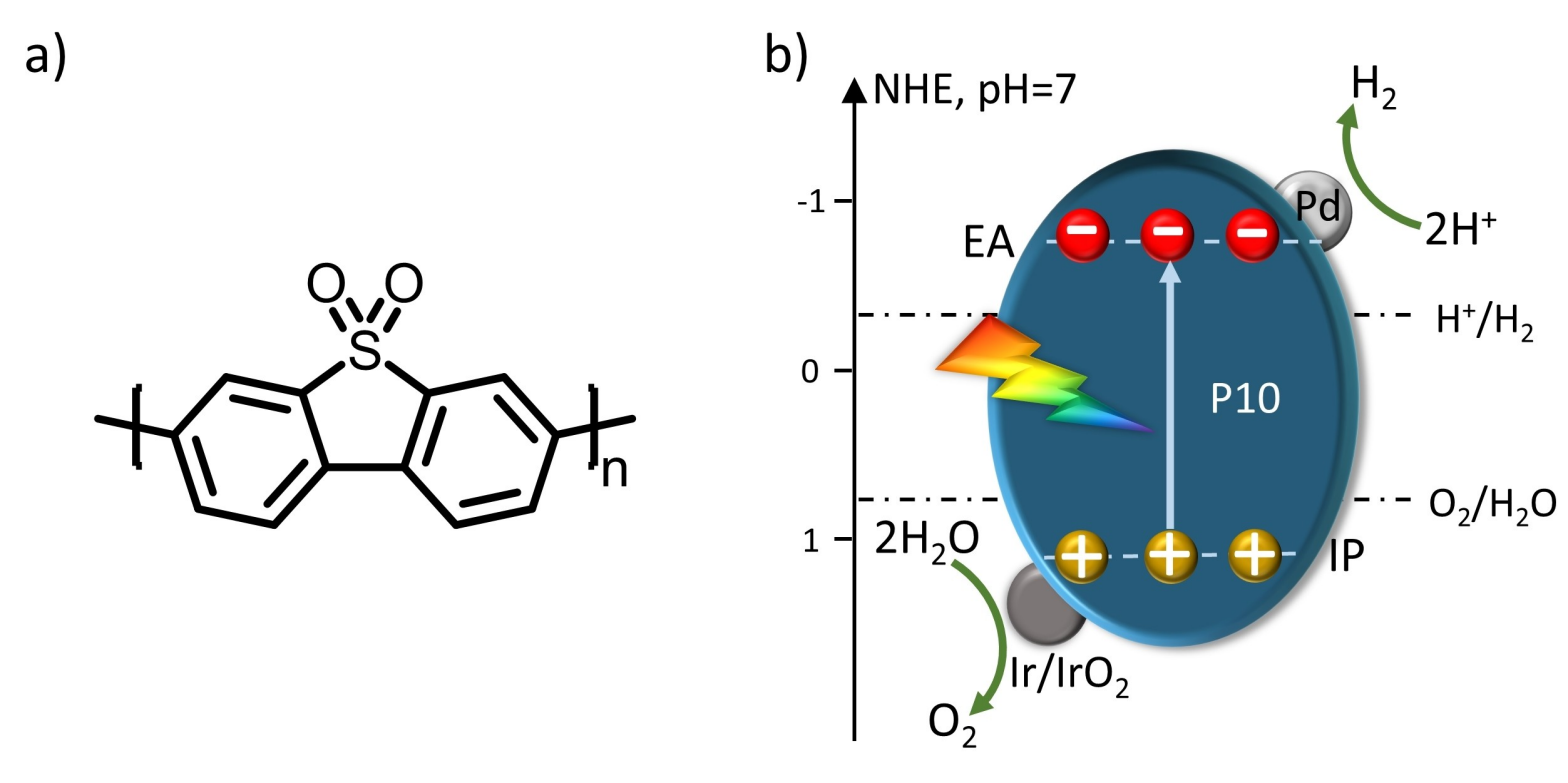
a) Chemical structure of the linear conjugated polymer photocatalyst P10. b) Alignment of the P10 energy levels (IP, ionization potential; EA, electron affinity) predicted by DFT relative to the potentials for proton reduction and water oxidation at pH 7. Underlying data taken from Ref. [38].

Single particulate polymer photocatalysts for overall water‐splitting that do not rely on redox mediators could overcome these limitations but they are rare. Two 1,3,5‐diyne‐linked conjugated microporous polymers were claimed to act as single component organic photocatalysts for overall water splitting,^[28]^ without any metal co‐catalysts. Most reported systems require a metal co‐catalyst to archive overall water splitting.[^27^, ^29^, ^30^] This is because metal co‐catalysts facilitate charge separation,^[31]^ store charges, and serve as reaction sites that catalyze water oxidation and reduction.[^24^, ^32^, ^33^] As such, much effort has been spent in the development of co‐catalysts for photocatalysis.[^34^, ^35^] For example, carbon nitride loaded with Pt/CoO_*x*_ as co‐catalyst was reported to be active for photocatalytic overall water splitting.^[36]^ Similarly, a covalent triazine‐based framework loaded with NiP_*x*_/Pt was reported to act as a single component photocatalyst for overall water splitting.^[37]^ Here, we explored the homopolymer of dibenzo[*b*,*d*]thiophene sulfone (P10, Figure 1a), which was shown previously to drive both proton reduction^[38]^ and water oxidation^[39]^ as separate half‐reactions in the presence of appropriate sacrificial electron donors or acceptors. The linear conjugated polymer P10 is also predicted to be able to drive overall water splitting (Figure 1b). Given the importance of co‐catalysts, we explored a range of metals loaded onto P10 for overall water splitting in absence of sacrificial reagents. Overall water splitting reaction was found to proceed by using P10 loaded with iridium (P10‐Ir) under optimized reaction conditions, which is the first example of single component photocatalyst for water splitting that uses a linear conjugated polymer. We then used transient spectroscopy to study the kinetics of the system and found that the co‐catalyst opens up new kinetic pathways for the system.

## Results and Discussion

**Figure 2:**
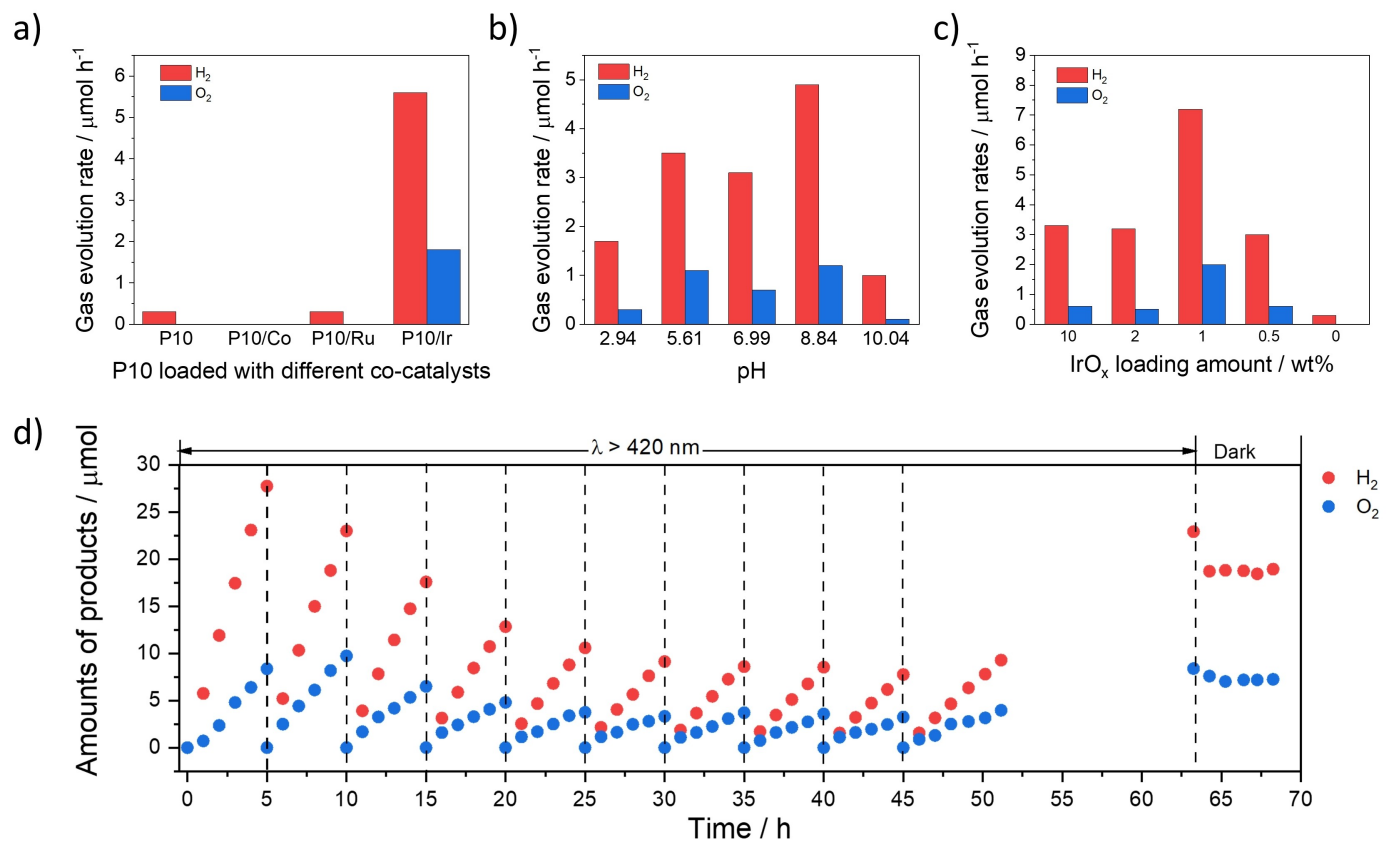
a) Dependence of gas evolution rates on the different co‐catalyst (1 wt. %) loaded onto P10 (1 mg) under visible light illumination. b) Effect of pH of reactant solution on photocatalytic water splitting over P10‐Ir (1 mg) under visible light (*λ*>420 nm), pH was adjusted using H_2_SO_4_ or KOH. c) Effect of loading amount of Ir cocatalyst on P10‐Ir on photocatalytic water splitting under visible light (*λ*>420 nm). d) Photocatalytic water splitting over P10‐Ir (1 mg) under visible light (*λ*>420 nm), the change of gas amount at 63 hours occurred because the reactor temperature changes after the light source was turned off. When left in the dark after the extended run the change in temperature results in an initial reduction of the measured gas products, but no further reduction of the amounts of H_2_ and O_2_ was observed, suggesting that no significant backward reaction was taking place. Experiments in a–d were carried out with PerkinElmer CERMAX PE300BF 300 W Xe light source with cut‐off filters, irradiation area: 33 cm^2^, *λ*>420 nm; top‐irradiation cell with a Pyrex window in a gas‐closed circulation system. Reactant solution: distilled water (120 mL). Activities were calculated from photocatalytic experiments without initial stabilization over 5 hours (a–c). See Ref. [2] for experimental set‐up used.

The photocatalyst P10 (the homopolymer of dibenzo[*b*,*d*]thiophene sulfone) was synthesized by Pd^0^‐catalyzed Suzuki–Miyaura cross‐coupling reaction and purified using Soxhlet extraction with chloroform. Characterization was found to match our previous reports of the material.[^31^, ^38^] P10 contains residual metallic palladium particles that act as a co‐catalyst for hydrogen production, as we have shown previously.^[31]^ The literature suggests that a second co‐catalyst would be required to facilitate simultaneous water oxidation, thus allowing for photocatalytic overall water splitting to take place. P10 was therefore further optimized by loading it with different co‐catalyst using a microwave heating method.^[40]^ The activity of photocatalyst P10 loaded with various co‐catalysts for overall water splitting is shown in Figure 2a. Cobalt was found to enable water oxidation with P10 in the presence of silver(I) nitrate acting as an electron scavenger,^[39]^ but P10 loaded with CoO_*x*_ was found to be inactive for overall water splitting. Ruthenium oxide has also been reported as a efficiency hydrogen evolution co‐catalyst,[^11^, ^41^] but it did not facilitate overall water splitting here, with only a small amount of hydrogen being produced without apparent oxygen production. IrO_2_ has also been reported as a co‐catalyst for overall water splitting.^[51]^ We found that IrO_2_ loaded P10, formed by addition of P10 to an [NH_4_IrCl_6_] aqueous solution prior to microwave heating and after irradiation in water, to be effective; overall water splitting proceeded under visible irradiation with initial rates of 5.6 μmol h^−1^ and 1.8 μmol h^−1^ for hydrogen and oxygen production under an experimental condition as shown in the caption of Figure 2. P10‐IrO_2_ was also tested for sacrificial oxygen evolution using aqueous AgNO_3_ solution as the scavenger. Under these conditions, it was found that the photocatalyst produces oxygen (Figure S‐3), unlike P10 without iridium loading,^[39]^ demonstrating the importance of the iridium in driving the water oxidation half reaction.

**Figure 3:**
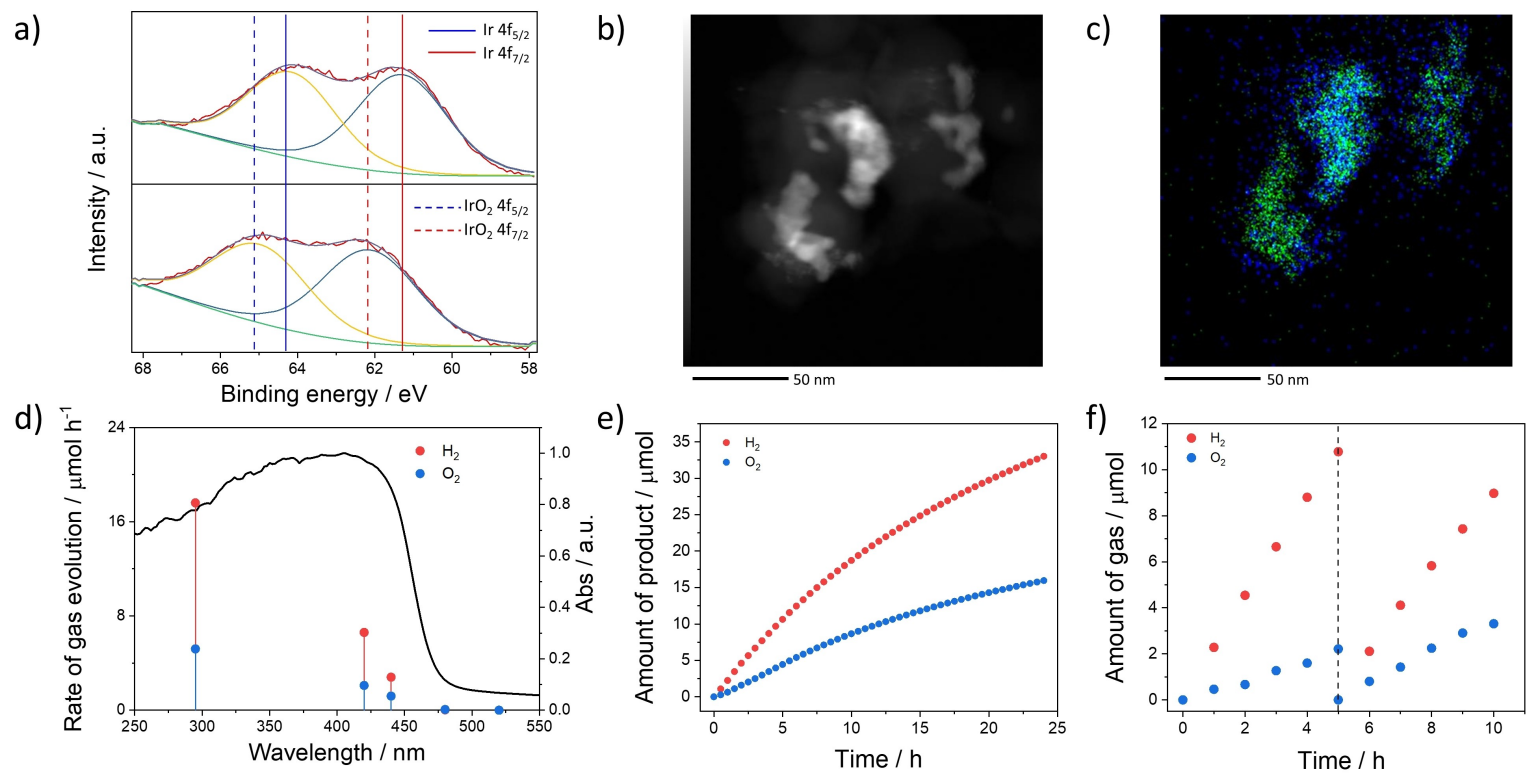
X‐Ray photoelectron spectra of a) P10‐Ir (top) and P10‐IrO_2_ (bottom). b) STEM‐HAADF image of the photocatalyst after photocatalysis (the scale bar is 50 nm long). c) EDX mapping of the same area as in b showing the presence of palladium (green) and iridium (blue) (the scale bar is 50 nm long). d) Wavelength dependence of photocatalytic water splitting over P10‐Ir (1 mg) in distilled water (120 mL) in gas‐closed circulation system, light source: 300 W Xe‐arc light source with different cut‐off filters, irradiation area: 33 cm^2^. e) Photocatalytic water splitting over P10‐Ir (1 mg) in distilled water (120 mL) in Ar‐flow system (1 atm) under visible light (*λ*>420 nm, 300 W, irradiation area: 33 cm^2^). f) Photocatalytic solar water splitting over P10‐Ir (1 mg) in distilled water (120 mL) in gas‐closed circulation system, light source: solar simulator with an AM1.5G filter (100 mW cm^−2^), irradiation area: 25 cm^2^ (see Ref. [2] for experimental set‐up used).

High‐resolution transmission electron microscopy showed that metal particles were evenly distributed throughout the polymer and identified to be iridium and palladium by energy‐dispersive X‐ray spectroscopy (EDX) mapping (Figure S‐27). In line with previous reports, we observed palladium particles sized between 10 and 15 nm[^26^, ^42^] while the iridium particles were approximately 2 nm in diameter. During photocatalysis iridium is converted to IrO_2_ as evident from XPS measurements (Figure 3a). It appears that the larger palladium particles are partially covered with IrO_2_, while also free small IrO_2_ particles exist (Figure 3b, c). Palladium is very likely acting as the proton reduction catalyst, but it is also well‐known to act as an electron‐hole recombination center in photocatalytic water splitting.[^43^, ^44^]

The photocatalytic activity of P10‐Ir was studied as a function of pH with IrO_2_ has been reported to be stable from pH 2–12.^[52]^ The performance was found to vary little in the pH range of 5.6–9.0, and activities were lower at pH 2.9 and pH 10 (Figure 2b). One possible explanation is the calculated ionization potential and electron affinity of the polymer relative to the potential of water reduction and oxidation (Figure S‐19). At low pH, the driving force (i.e., the difference between the water oxidation potential and the ionization potential of the polymer) is small compared to higher pH, while at the other extreme (pH 10), the difference between the proton reduction potential and the electron affinity of the polymer is smaller compared to that at lower pH. It appears, therefore, that pH values close to pH 7 offer sufficient driving force for both half reactions and therefore the highest photocatalytic activities. The water splitting activity of P10‐IrO_2_ was not increased by using larger amounts of P10‐IrO_2_ in water with the hydrogen evolution rate of 50 mg P10‐IrO_2_ only slightly increasing to 10.9 μmol h^−1^ compared to experiments using 1 mg P10‐IrO_2_ (5.6 μmol h^−1^) as shown in Table 1. More significantly, there was no measured increase in the amount of oxygen produced, suggesting that the increase in hydrogen originated from either decomposition of impurities or auto‐oxidation of the photocatalyst.

**Table 1:** Photocatalytic water splitting under visible light illumination (λ>420 nm) for particulate conjugated polymer P10 in water.

Entry	Amount of P10^[a]^ [mg]	Co‐catalyst (wt. %)	H_2_ Evolution rate [μmol h^−1^]^[b]^	O_2_ Evolution rate [μmol h^−1^]^[b]^	Kinetic data
1	50	Ir (0.45)	10.9	0.7	Figure S‐4
2	10	Ir (0.45)	7.4	1.4	Figure S‐5
3	5	Ir (0.45)	6.6	2.1	Figure S‐6
4	3	Ir (0.45)	6.4	2	Figure S‐7
5	1	Ir (0.45)	5.6	1.8	Figure 2d
6	1	Co (0.04)	0	0	–
7	1	Ru (0.8)	0.3	0	Figure S‐16
8	5	–^[c]^	0.3	0	Figure S‐17
9	1	Ir (0.45)	2.1^[d]^	0.9^[d]^	Figure 2d

[a] Reaction conditions: P10 containing Pd loaded with additional Ir, Co or Ru by microwave deposition (details in Supporting Information); 300 W Xe light source with a cut‐off filter (*λ*>420 nm); cell, top‐irradiation, 70 torr, Ar, reactant solution: distilled water. [b] Gas evolution rates were calculated from the first run of the photocatalytic experiments. [c] No additional co‐catalyst was added. [d] After 20 hours equilibration.

We also tried to optimize the amount of Ir loaded onto P10 as shown in Figure 2c. In the absence of iridium no photocatalytic overall water splitting is observed, and only a small amount of hydrogen is detected, which we have observed previously and can potentially be ascribed to the decomposition of impurities or auto‐oxidation of the photocatalyst (Figure S‐17).[^18^, ^38^] A loading of 1 % Ir on P10 gave the highest activity for water splitting with higher loadings (2 % and 10 %) reducing the activity, possibly due to parasitic light absorption of the metal that competes with the polymer photocatalyst. IrO_2_ on its own was found to be inactive under broadband irradiation in pure water, ruling out that it acts as a photocatalyst on its own.

The initial photocatalytic evolution production rate of 5.6 μmol h^−1^ for hydrogen and 1.8 μmol h^−1^ for oxygen for P10‐IrO_2_ (1 % Ir loading, 1 mg, 120 mL water, 300 W Xe light source, *λ*>420 nm) decreased over each of the subsequent 5 hour runs before stabilizing to constant water splitting at rates of 2.1 μmol h^−1^ for hydrogen and 0.9 μmol h^−1^ for oxygen production. The H_2_/O_2_ production ratio is close to 2, within an experimental error after the stabilization period (Figure 2d). Similar observations of a stabilization period have been made previously for germanium nitride loaded with RuO_2_ nanoparticles.^[45]^ This might be caused by the oxidation of the iridium in P10‐Ir to IrO_2_ (Figure 3a) as evident from XPS measurements showing that metallic iridium is converted into IrO_2_ during light irradiation (Figure 3a)—or by oxidation of residual compounds such as ethylene glycol used for the Ir‐loading step as might indicated by the increased hydrogen evolution and reduced oxygen evolution. Detachment of the Ir species from the surface of P10‐IrO_2_ during the early stages of the catalysis could also be a possible reason since the content of Ir in P10‐IrO_2_ (1 % Ir loading) was reduced from 0.45 % to 0.35 % after the experiment (Table S‐1).

The total amount of hydrogen evolved was 148.7 μmol over 63 hours, which was larger than the amount of hydrogen in P10‐Ir sample (14 μmol). The turnover number was determined to be 54.1 after 63 hours per mole of repeat unit of P10 based on a 4‐hole hole transfer to water resulting in oxygen production [see Supporting Information for calculations, Eq. (S‐3)]. The amount of oxygen produced (55.4 μmol) is far greater than the oxygen content of the polymer used in the experiment (4 μmol), further ruling out polymer decomposition to be responsible for oxygen production. Post illumination analysis also showed no significant changes in the UV/Vis, photoluminescence, FT‐IR spectra, and PXRD patterns for the catalysts (Figure S‐41 to S‐44). These results clearly demonstrated that P10‐Ir is a photocatalyst suitable for overall water splitting even when other reports indicate that sustained water oxidation is difficult to maintain for organic materials.[^39^, ^46^]

P10‐Ir showed higher rates of 17.6 μmol h^−1^ and 5.2 μmol h^−1^ for H_2_ and O_2_ production under broadband illumination (full arc, 300 W Xe light source, 70 torr) when compared to visible (*λ*>420 nm) light alone (H_2_: 5.6 μmol h^−1^ and O_2_: 1.8 μmol h^−1^). This is expected as the photocatalyst P10 absorbs both UV and visible photons (Figure 3d). The rates of hydrogen and oxygen evolution are reduced to 2.8 μmol h^−1^ and 1.2 μmol h^−1^ with a 440 nm long‐pass cut‐off filter, only 0.03 μmol h^−1^ and 0.06 μmol h^−1^ with a 480 nm filter, and no photocatalytic activity was observed when using a 520 nm cut‐off filter (300 W, Xe light source; 70 torr). This shows that the efficiency tracks the absorption profile of P10‐IrO_2_ and that process is driven by the absorption of light.

The experiments described above were performed under reduced pressure, which allows for both hydrogen and oxygen gas to be driven off the surface. To test the activity of P10‐Ir under atmospheric pressure experiments were performed in a flow system with an argon carrier gas (flow rate=15 mL min^−1^). These experiments demonstrated that under visible irradiation (*λ*>420 nm) P10‐IrO_2_ also produces H_2_ and O_2_ from water at ambient pressure (Figure 3e) with lower but measurable rates (H_2_: 1.36 μmol h^−1^, O_2_: 0.66 μmol h^−1^) in a 2.06 : 1 ratio. Under a solar simulator, we observed rates of 1.8 μmol h^−1^ for H_2_, and 0.7 μmol h^−1^ for O_2_ after 5 hours stabilization (Figure 3f).

Using monochromatic light, we attempted to measure the apparent quantum efficiency (AQY) of the photocatalytic system. Using 350 nm irradiation an AQY of 0.062 % [Eq. (S‐7)] for hydrogen production was determined, however, the water splitting reaction yielded non‐stoichiometric gas evolution (Figure S‐20).

Finally, again using a solar simulator, the solar‐to‐hydrogen efficiency (STH) was determined to be 0.0047 % [Eq. (S‐6)]. This is a more than 3 times improvement compared to our previous report of P10 in a Z‐scheme with BiVO_4_ (0.0014 %).^[26]^ The low STH efficiency can be explained, in part, by the low polymer loading: much of the light passes through the reactor without being absorbed. Inorganic photocatalysts, such as aluminum‐doped strontium titanate loaded with Rh/Cr_2_O_3_/CoOOH have shown much higher STH values of 0.65 %.^[12]^ However, we note that the catalytic activity for sacrificial hydrogen evolution in organic polymers increased by a factor of 600 in the period 2015 (pyrene networks)^[17]^ to 2020 (bulk heterojunction materials)[^47^, ^48^] and thus far, only a very small number of polymer photocatalysts have been reported for overall water splitting.

**Figure 4:**
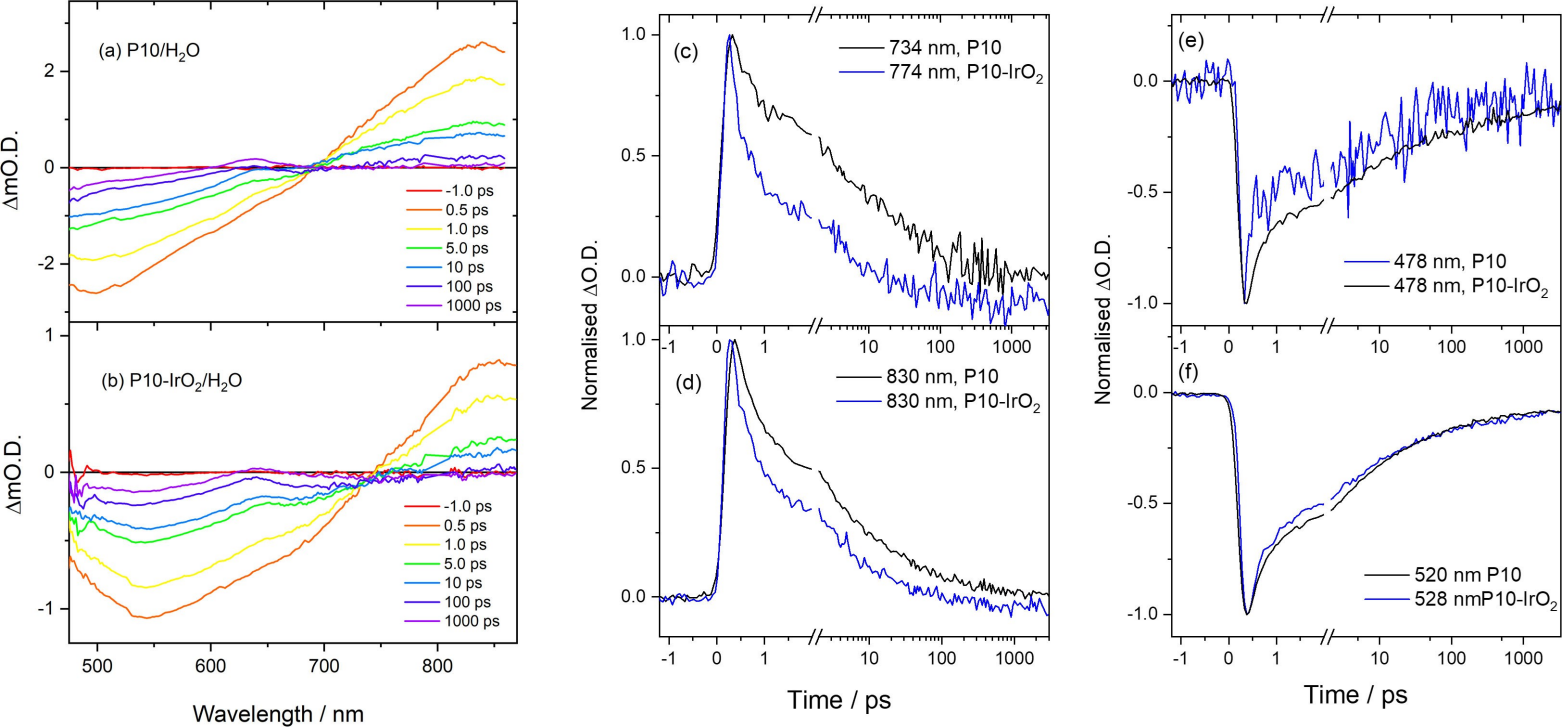
a) TA difference spectra of P10 and b) P10‐IrO_2_ (post photocatalysis) in water following 400 nm excitation. c) and d) kinetic traces showing the decay of the photoinduced absorption of P10 and P10‐IrO_2_ at wavelengths that the global fitting identified as maxima in the species associated spectra. e) and f) kinetics of the stimulated emission of P10 and P10‐IrO_2_.

Transient absorption (TA) UV/Vis spectroscopy can provide evidence of the mechanism of photocatalysis and was used previously to study the formation and fate of the P10 electron polaron during hydrogen[^31^, ^38^] and oxygen^[39]^ evolution, as well as in a Z‐scheme device.^[26]^ Excitation at 400 nm of P10 and P10‐IrO_2_ (post‐photocatalysis) in water under an Ar atmosphere leads to similar spectra on the ps‐ns timescales, Figure 4. The broad positive absorption peaking at ca. 870 nm has been assigned elsewhere to excited state absorption by a singlet exciton state,^[38]^ which we show below is actually composed of spectral features of 2 or more states. These excitonic states can radiatively decay back to the ground state, with stimulated emission giving rise to the negative features, with the spectral position coinciding with the broad band observed in the steady‐state emission spectra of P10 and P10‐IrO_2_ suspensions (Figure S‐35). Alternatively, a long‐lived polaronic state can be formed that has an absorption maximum at 637 nm (P10) or 635 nm (P10‐IrO_2_). The assignment of this TA band to a polaronic state is based on past experiments where in the presence of a sacrificial electron donor a P10 electron polaron was found to persist for hundreds of microseconds prior to electron transfer to the Pd HER catalyst.^[31]^ In the absence of a sacrificial electron donor, similar spectral features are typically assigned to a polaron pair.^[38]^ The presence of IrO_2_ accelerates the decay of the broad positive absorption assigned to the P10 excitonic states (Figure 4c, d), with the difference clearly noticeable at times <2 ps. In contrast the rate of recovery of the negative bleach assigned to stimulated emission shows no clear dependence on IrO_2_ on the ps‐ns timescale (Figure 4e, f). Therefore, either an additional non‐radiative decay pathway becomes available, or an acceleration of an existing pathway occurs, when IrO_2_ is present in P10.

**Figure 5:**
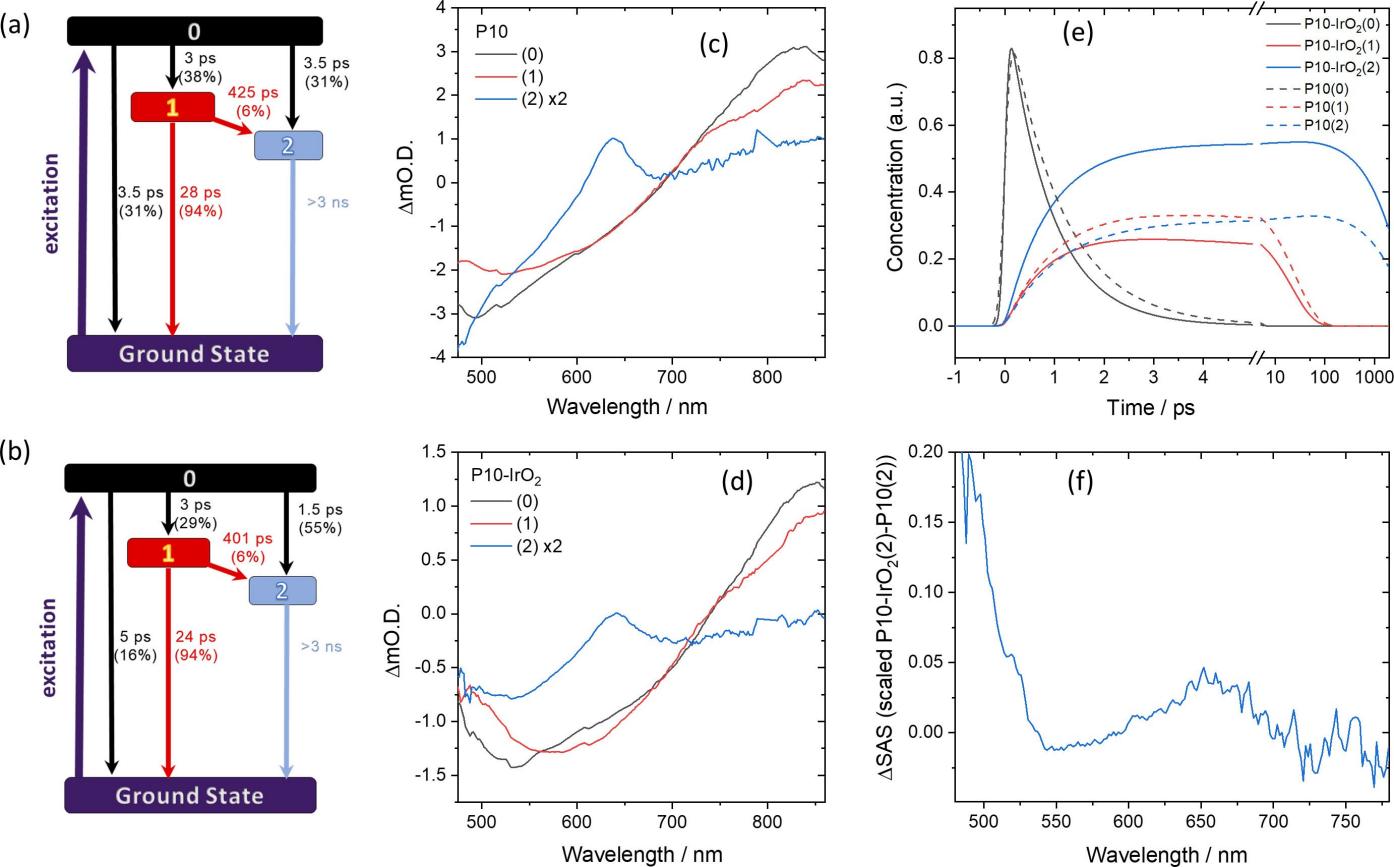
a) Kinetic model used for the global target analysis of the TA spectra of P10 and b) P10‐IrO_2_ following 400 nm excitation which shows accelerated formation of the P10 polaron species (2) in the presence of the IrO_2_ which is the charge separated state involved in photocatalysis. c) SAS of components 0, 1, 2 generated for P10 and d) P10‐IrO_2_. e) Calculated amplitudes of the individual species with time. The dashed lines represent P10 and the solid lines P10‐IrO_2_. f) Difference in SAS of component 2 of P10‐IrO_2_ and P10 indicating hole transfer to the IrO_2_ co‐catalyst. The relative intensity of the spectra was scaled until the ≈635 nm P10^(−)^ peak was not observable in the difference spectra.

To identify this pathway and to obtain the spectral fingerprints of individual components from the complex overlapping spectra, global target analysis was carried out on both data sets. Full details of the procedure and the global lifetime approach, which requires fewer assumptions but still provides similar conclusions, are given in the Supporting Information. The kinetic model consists of three states, an initially generated excited state (0) which can populate two lower energy states (1,2). Initial fitting with a 2‐compartment mode consisting solely of an initially generated excitonic state and a charge separated state gave a poor fit to the experimental data. Based on the global lifetime analysis, we allowed transfer of population from compartment 0 to 1 and 2, and from 1 to 2. In addition, all 3 compartments were allowed to relax to the ground state (Figure 5a, b). The lifetimes and relative yield of each kinetic pathway are shown in Figure 5a, b, e.

The species associated spectra (SAS) generated in the global fitting procedure for P10 and P10‐IrO_2_ are shown in Figure 5c, d. Species 0 and 1 both have characteristic features of the singlet excitonic state. Given this observation, along with the identical time that species 1 takes to form in the presence and absence of IrO_2_, it can be assigned to a singlet excitonic state, which likely forms following vibrational relaxation of the initially formed hot state (species 0) in‐line with a recent time‐resolved Raman study of P10 which showed vibrational cooling occurs within 10 ps and that polaron formation can occur from both the vibrationally hot and thermalized exciton.^[53]^ The SAS of species 2 (P10) has a maximum at ca. 635 nm which agrees with the previously reported polaron pair and electron polaron spectra of P10. Interestingly the P10‐IrO_2_ SAS of species 2 shows a broadening of the 635 nm peak and an increasing ΔO.D. below 550 nm when compared to the P10 SAS of species 2. Subtracting the SAS of the polaronic states of P10 from P10‐IrO_2_ gives rise to the spectrum shown in Figure 5f. The difference in the SAS spectra of P10 and P10‐IrO_2_ is proposed to be due to rapid hole transfer to the IrO_2_ giving rise to a charge separated P10^(−)^‐IrO_2_^(+)^ state (2), which goes on to enables water oxidation at the IrO_2_ catalyst and hydrogen evolution via electron transfer to the Pd co‐catalyst. We note a good agreement between the feature present in Figure 4a and the UV/Vis spectrum reported of oxidized IrO_2_ obtained through spectroelectrochemistry, supporting this assignment.^[49]^ Our modelling of the TA data indicates the primary pathway for formation of the polaronic states of both P10 and P10‐IrO_2_ is directly from the initially generated hot exciton state (0). Relaxation into the lower energy excitonic state (1) represents a loss pathway with it primarily decaying to the ground state (94 %). The yield and rate of formation of the polaron (2) is greater with the P10‐IrO_2_ sample (Figure 5e), which rationalizes the decreased lifetime of the photoinduced absorption at *λ*>700 nm (Figure 5c, d). It is striking that the lifetime of formation of P10^(−)^‐IrO_2_^(+)^ is estimated to be only 1.5 ps allowing it to compete with the kinetics of recombination, which enables water oxidation using a single polymer photocatalyst.

Besides P10, a number of other linear conjugated polymers are known experimentally to drive both proton reduction and water oxidation in the presence of sacrificial donors^[39]^ and many more should theoretically be able to drive overall water splitting based on their predicted ionization potential and electron affinity.^[20]^ The results presented here for P10 suggest that many of these polymers might be active for overall water splitting in the presence of suitable co‐catalysts and that such polymer photocatalysts can be relatively stable for this reaction under the right operating conditions.

## Conclusion

In summary, iridium‐loaded on P10 was found to be a co‐catalyst for the decomposition of H_2_O into H_2_ and O_2_, representing the first successful example of an organic photocatalyst for overall water splitting based on a linear conjugated polymer. Overall water splitting of P10 loaded with IrO_2_ co‐catalyst proceeded steadily for an extended period of time (>60 hours). The photocatalytic activity was strong dependent on the co‐catalysts with only Ir co‐catalyst found to drive overall water splitting for the P10 photocatalyst. Transient absorption UV/Vis spectroscopy was used to study the photocatalytic system and species associated spectra analyzed were generated. The analysis suggest that a charge separated P10(^−^)‐IrO_2_(^+^) state is formed rapidly that enables water oxidation at the IrO_2_ catalyst and hydrogen evolution via electron transfer to the Pd co‐catalyst, highlighting the importance of the Ir‐cocatalyst. Although the overall solar‐to‐hydrogen efficiency of this first system is very low with respect to inorganic semiconductors, as for the first embodiments of polymers for sacrificial hydrogen production,^[17]^ it provides proof‐of‐concept study that linear polymer photocatalysts can in principle move away from using sacrificial reagents for hydrogen production.^[50]^

## Conflict of interest

The authors declare no conflict of interest.

1

## Supporting information

As a service to our authors and readers, this journal provides supporting information supplied by the authors. Such materials are peer reviewed and may be re‐organized for online delivery, but are not copy‐edited or typeset. Technical support issues arising from supporting information (other than missing files) should be addressed to the authors.

Supporting Information

## Data Availability

The data that support the findings of this study are available in the Supporting Information of this article.
