# Supplementary material for: Photocatalytic Overall Water Splitting Under Visible Light Enabled by a Particulate Conjugated Polymer Loaded with Palladium and Iridium
Source: Angew Chem Int Ed Engl. 2022 Apr 26;61(26):e202201299. doi: 10.1002/anie.202201299 (PMC9321674; doi:10.1002/anie.202201299)
Supplement: Supplementary file 1 — Supporting Information [file ANIE-61-0-s001.pdf]

Angewandte Chemie

Eine Zeitschrift der Gesellschaft Deutscher Chemiker

Supporting Information

Photocatalytic Overall Water Splitting Under Visible Light Enabled by a Particulate Conjugated Polymer Loaded with Palladium and Iridium

Y. Bai, C. Li, L. Liu, Y. Yamaguchi, M. Bahri, H. Yang, A. Gardner, M. A. Zwijnenburg, N. D. Browning, A. J. Cowan, A. Kudo*, A. I. Cooper*, R. S. Sprick**

Supplementary information

for

Photocatalytic Overall Water Splitting Under Visible Light Enabled by a Particulate Conjugated Polymer Loaded with Palladium and Iridium

Yang Bai,[1,2] Chao Li,[3] Lunjie Liu,[1] Yuichi Yamaguchi,[4] Mounib Bahri,[5] Haofan Yang,[1] Adrian Gardner,[3] Martijn A. Zwijnenburg,[6] Nigel D. Browning,[5] Alexander J. Cowan,[3,*] Akihiko Kudo,[4,*] Andrew I. Cooper[1,*] and Reiner Sebastian Sprick,[1,7,*]

[1] Materials Innovation Factory & Department of Chemistry, University of Liverpool, 51 Oxford Street, Liverpool L7 3NY, UK.

[2] Institute of Materials Research and Engineering, Agency for Science Technology and Research, #08-03, 2 Fusionopolis Way, Innovis, Singapore, 138634, Singapore.

[3] Stephenson Institute for Renewable Energy, University of Liverpool, Peach Street, Liverpool L69 7ZF, UK.

[4] Tokyo University of Science, Department of Applied Chemistry, 1-3 Kagurazaka, Shinjuku-ku, Tokyo, Japan

[5] Albert Crewe Centre for Electron Microscopy, University of Liverpool, Liverpool L69 3GL, UK.

[6] Department of Chemistry, University College London, 20 Gordon Street, London WC1H 0AJ, UK.

[7] Department of Pure and Applied Chemistry, University of Strathclyde, Thomas Graham Building, 295 Cathedral Street, Glasgow G1 1XL, UK.

Reagents and solvents were purchased from commercial suppliers (Manchester Organics, Sigma-Aldrich, Kanto Chemical, Wako Pure Chemical Industries, and Tanaka Kikinzoku) and used without further purification. P10 was synthesized using a previously reported method.[1,2] FT-IR spectra were recorded on a Bruker Tensor 27 with an ATR attachment at room temperature. UV-Visible spectra of the polymers were recorded on a Shimadzu UV-2550 UV-Vis spectrometer as powders in the solid state. Photoluminescence spectra of the polymer powders were measured with a Shimadzu RF-5301PC fluorescence spectrometer at room temperature. Time-correlated single photon counting (TCSPC) experiments were performed on an Edinburgh Instruments LS980-D2S2-STM spectrometer equipped with picosecond pulsed LED excitation sources and a R928 detector, with a stop count rate below 5%. An EPL-375 diode (λ = 370.5 nm, instrument response 100 ps, fwhm) was used. Suspensions were prepared by ultrasonicating the polymer in water. The instrument response was measured with colloidal silica (LUDOX® HS-40, Sigma-Aldrich) at the excitation wavelength. Decay times were fitted in the FAST software using suggested lifetime estimates. PXRD measurements were performed on a PANalytical X'Pert PRO MPD, with a Cu X-ray source, used in high throughput transmission mode with Kα focusing mirror and PIXCEL 1D detector. Static light scattering measurements were performed on a Malvern Mastersizer 3000 Particle Sizer. Sample was dispersed in water by 10 minutes of ultrasonication and the resultant suspensions were injected into a stirred Hydro SV quartz cell, containing more water, to give a laser obscuration of 5 – 12%. Particle sizes were fitted according to Mie theory, using the Malvern 'General Purpose' analysis model, for non-spherical particles. A sample refractive index of 1.59, sample absorbance of 0.1 and solvent refractive index of 1.333 were used for fitting. Inductively coupled plasma optical emission spectrometry (ICP-OES) analysis was performed on an ICP-OES Agilent 5110 with equipped with a collision/reaction cell after a microwave digest of the materials in nitric acid (67-69%, trace metal analysis grade) in a microwave. The solutions were diluted with water before the measurement and the instrument was calibrated with Pd and Ru standards in aqueous solution and Y-89 as the internal standard. HR-STEM observations were performed using a spherical aberration-corrected JEOL 2100 F operating at 200 kV. EDS mapping was performed using JEOL Silicon Drift Detector (DrySD100GV: with a solid angle of up to 0.98 steradians from a detection area of 100 mm^2).

Loading of Ir on P10

Table S-1. Varied amounts iridium loaded onto photocatalyst **P10**.

Polymer / Loaded amount of Ir	Measured Ir content / wt. %[a]
**P10**	0
**P10-Ir (0.5% Ir)**	0.20
**P10-Ir (1% Ir)**	0.45
**P10-Ir (2% Ir)**	0.61
**P10-Ir (10% Ir)**	1.33
**P10-IrO_2 (P10-Ir (1% Ir)-after photocatalysis)**	0.35

[a] The amount of loading iridium in the material as measured via ICP-OES.

Loading of Ir onto P10

P10 (0.2 g) was dispersed in ethylene glycol (40 mL) followed by the addition of an appropriate amount of an [NH_4IrCl_6] aqueous solution (2 wt. % Ir, calculated based on elemental Ir). The mixture was transferred into a glass vial and heated in a microwave reactor (Monowave 300, Anton Paar Ltd.) at 423 K for 30 minutes. Yielding the Ir loaded P10 photocatalyst as fine particles.

Loading of Co onto P10

P10 (0.2 g) was dispersed in ethylene glycol (40 mL) followed by the addition of aqueous $CoCl_3$ solution (80 mM, 106 µL). The mixture was transferred into a glass reactor and heated in a microwave reactor (Monowave 300, Anton Paar Company) at 150 °C for 30 minutes. Pd content: 0.11 wt. %, Co content: 0.04 wt. % as determined *via* ICP-OES.

Loading of Ru onto P10

P10 (0.2 g) was dispersed in ethylene glycol (40 mL) followed by the addition of aqueous $RuCl_3$ (37.5 mM, 198 µL). The mixture was transferred into a glass reactor and heated in a microwave reactor (Monowave 300, Anton Paar Company) at 150 °C for 30 minutes. Pd content: 0.11 wt. %, Ru content: 0.80 wt. % as determined via ICP-OES.

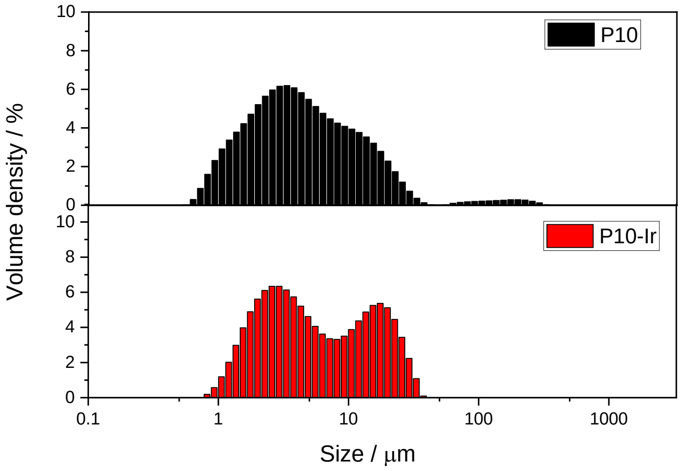

Figure S-1. Static light scattering experiments of polymers in water.

Table S-2. Particle sizes by static light scattering.

**Polymer**	***D*x50[a] / µm**	***D*[4,3][b] / µm**	***D*[3,2][c] / µm**	**Relative external surface area[d] / m^2 kg^{-1}**
**P10**	4.08	9.79	2.98	2012
**P10-Ir**	4.93	8.56	3.77	1591

[a] 50th percentile of particle size volume distribution; [b] Volume mean diameter; [c] Surface area mean diameter (Sauter mean diameter);[5,6] [d] Relative extrinsic surface area calculated by dividing the total surface area of the particles by the total mass, assuming a density of 1 g cm^{-3}.

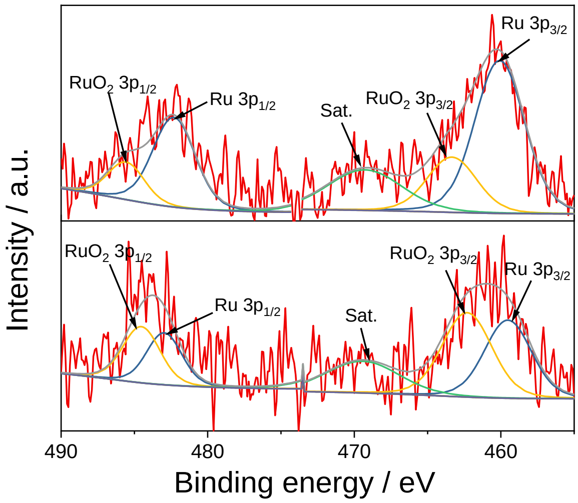

Figure S-2. X-Ray photoelectron spectra of a) P10-Ru before photocatalysis (top) and P10-Ru after photocatalysis (bottom).

Water splitting experiments were carried out in gas-closed circulation and Ar-flow systems. The P10-Ir (0.05-0.001 g) photocatalyst powder was dispersed in distilled water (120 mL) in a reaction cell made of Pyrex glass. A top-irradiation cell with a Pyrex window was used after degassing by applying vacuum and purging with argon. The set-up was brought back to reduced pressure (70 torr) and irradiated with a 300 W Xe arc light source (PerkinElmer; CERMAX PE300BF) or a solar simulator (Yamashita Denso; YSS-80QA, 100 mW cm^{-2}). The measurement of wavelength dependency was carried out using a 300 W Xe-arc light source with cut-off filters (HOYA). Amounts of evolved hydrogen and oxygen were determined using an online gas chromatograph (Shimadzu; GC-8A, MS-5Å column, TCD, Ar carrier).

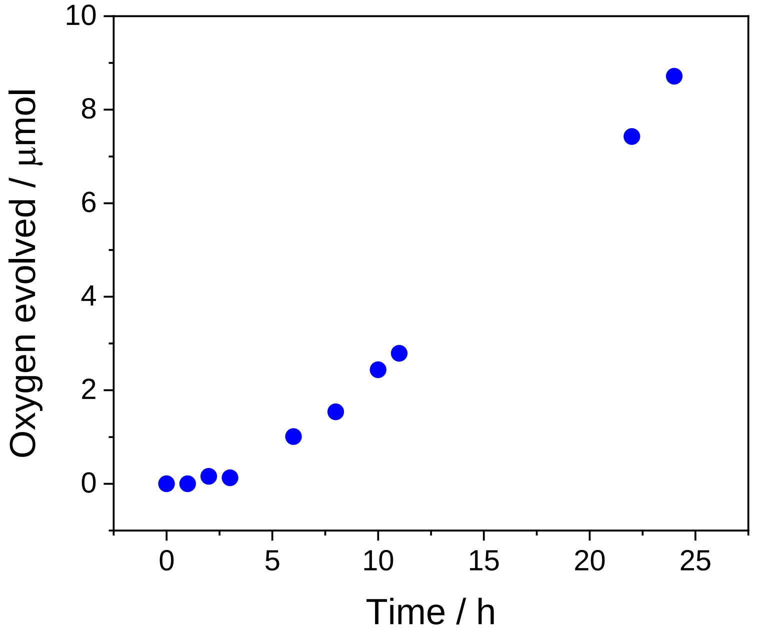

Figure S-3. Photocatalytic oxygen evolution over **P10-Ir** (50 mg) from an aqueous $AgNO_3$ solution (170 mg $AgNO_3$, 100 mL), pH was adjusted by La_2O_3 (200 mg). The reactions were carried out under visible light illumination (PerkinElmer CERMAX PE300BF 300 W Xe light source, λ > 420 nm) in a top-irradiation cell with a Pyrex window.

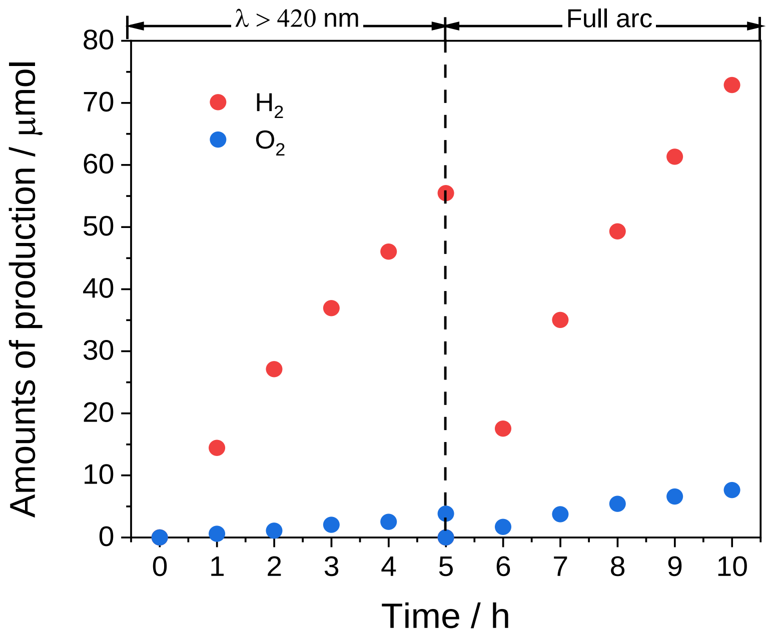

Figure S-4. Photocatalytic water splitting over **P10-Ir** (50 mg) in distilled water (120 mL) in gas-closed circulation system. light source: Xe light source (PerkinElmer CERMAX PE300BF 300 W Xe light source) with suitable cut-off filters in a top-irradiation cell with a Pyrex window, irradiation area: 33 cm^2.

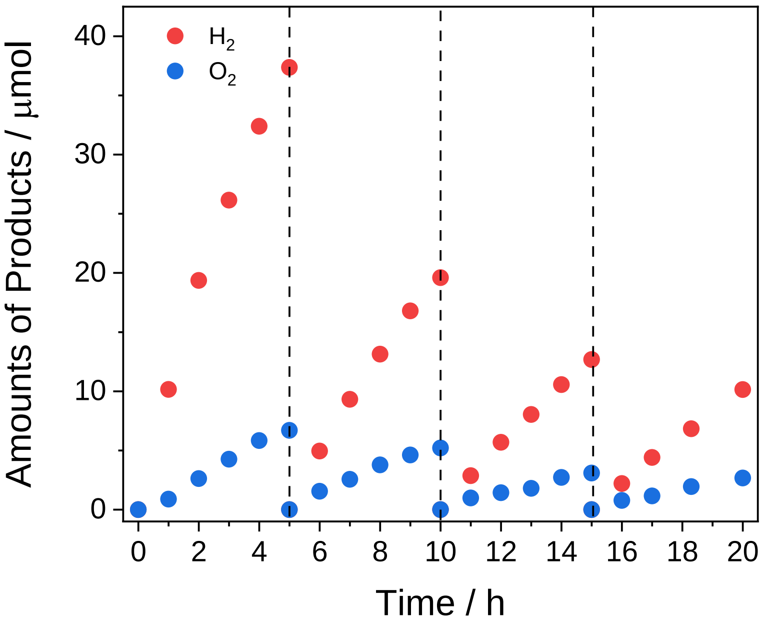

Figure S-5. Photocatalytic water splitting over **P10-Ir** (10 mg) in distilled water (120 mL) in gas-closed circulation system. light source: Xe light source (PerkinElmer CERMAX PE300BF 300 W Xe light source) with a 420 nm cut-off filter in a top-irradiation cell with a Pyrex window, irradiation area: 33 cm^2.

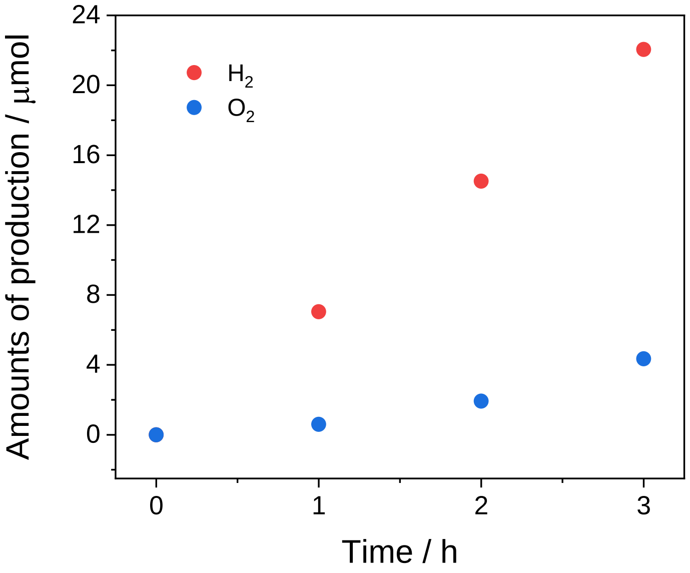

Figure S-6. Photocatalytic water splitting over **P10-Ir** (5 mg) in distilled water (120 mL) in gas-closed circulation system. light source: Xe light source (PerkinElmer CERMAX PE300BF 300 W Xe light source) with a 420 nm cut-off filter in a top-irradiation cell with a Pyrex window, irradiation area: 33 cm^2.

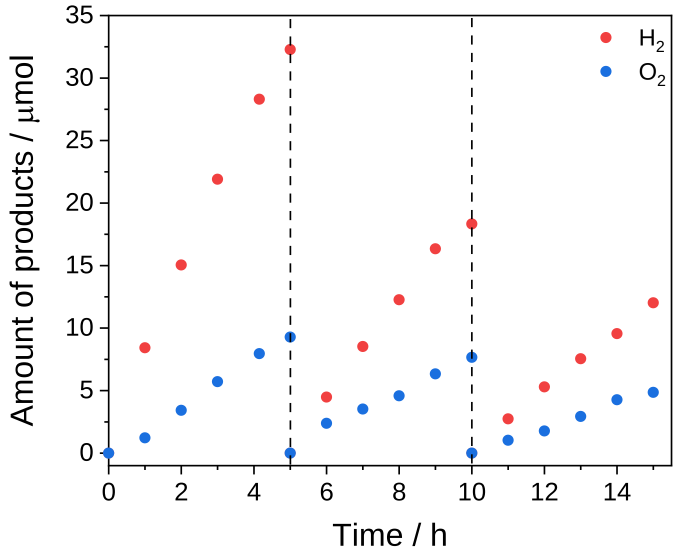

Figure S-7. Photocatalytic water splitting over **P10-Ir** (3 mg) in distilled water (120 mL) in gas-closed circulation system. light source: Xe light source (PerkinElmer CERMAX PE300BF 300 W Xe light source) with a 420 nm cut-off filter in a top-irradiation cell with a Pyrex window, irradiation area: 33 cm^2.

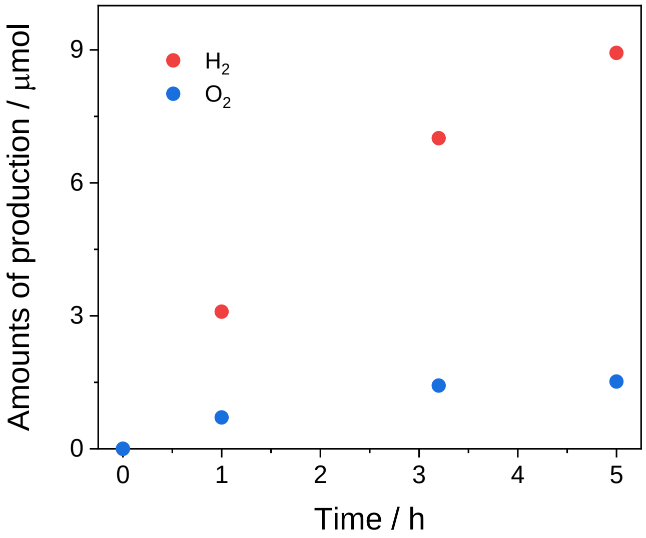

Figure S-8. Photocatalytic water splitting over **P10-Ir** (3 mg) in water (120 mL, pH 2.94, pH was adjusted by H_2SO_4) in gas-closed circulation system. light source: Xe light source (PerkinElmer CERMAX PE300BF 300 W Xe light source) with a 420 nm cut-off filter in a top-irradiation cell with a Pyrex window, irradiation area: 33 cm^2.

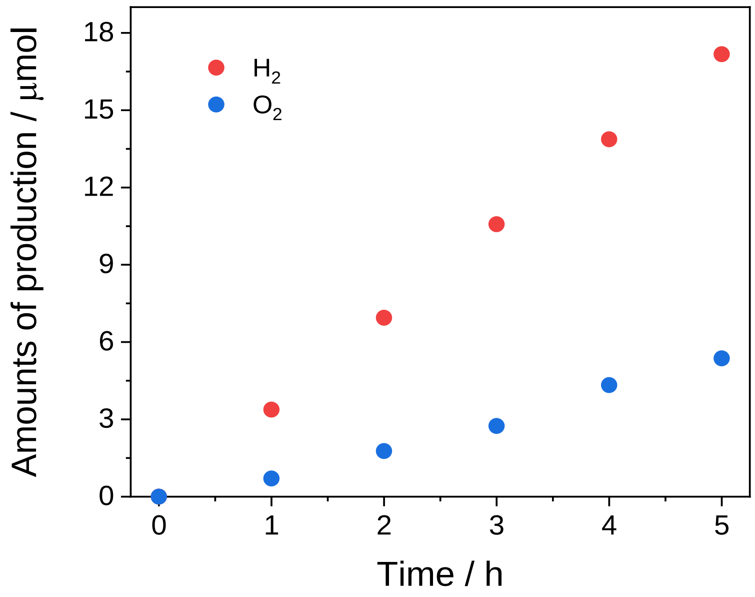

Figure S-9. Photocatalytic water splitting over **P10-Ir** (3 mg) in water (120 mL, pH: 5.61, neutral) in gas-closed circulation system. light source: Xe light source (PerkinElmer CERMAX PE300BF 300 W Xe light source) with 420 nm cut-off filter in a top-irradiation cell with a Pyrex window, irradiation area: 33 cm^2.

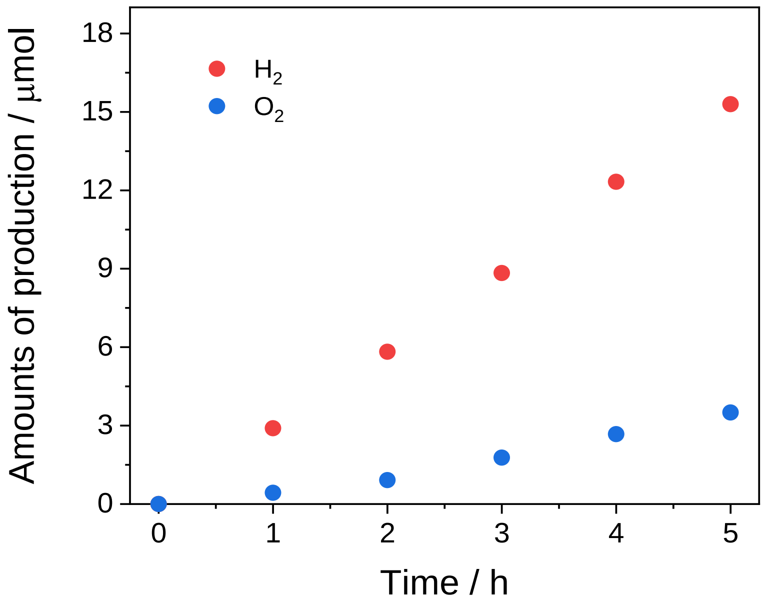

Figure S-10. Photocatalytic water splitting over **P10-Ir** (3 mg) in water (120 mL, pH 6.99, pH was adjusted by KOH) in gas-closed circulation system. light source: Xe light source (PerkinElmer CERMAX PE300BF 300 W Xe light source) with 420 nm cut-off filter in a top-irradiation cell with a Pyrex window, irradiation area: 33 cm^2.

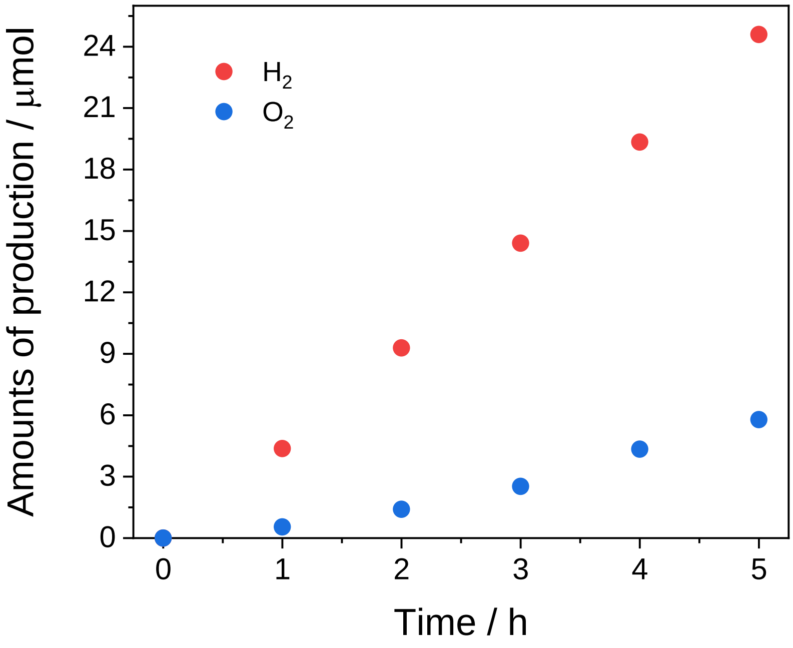

Figure S-11. Photocatalytic water splitting over **P10-Ir** (3 mg) in water (120 mL, pH 8.84, pH was adjusted by KOH) in gas-closed circulation system. light source: Xe light source (PerkinElmer CERMAX PE300BF 300 W Xe light source) with 420 nm cut-off filter in a top-irradiation cell with a Pyrex window, irradiation area: 33 cm^2.

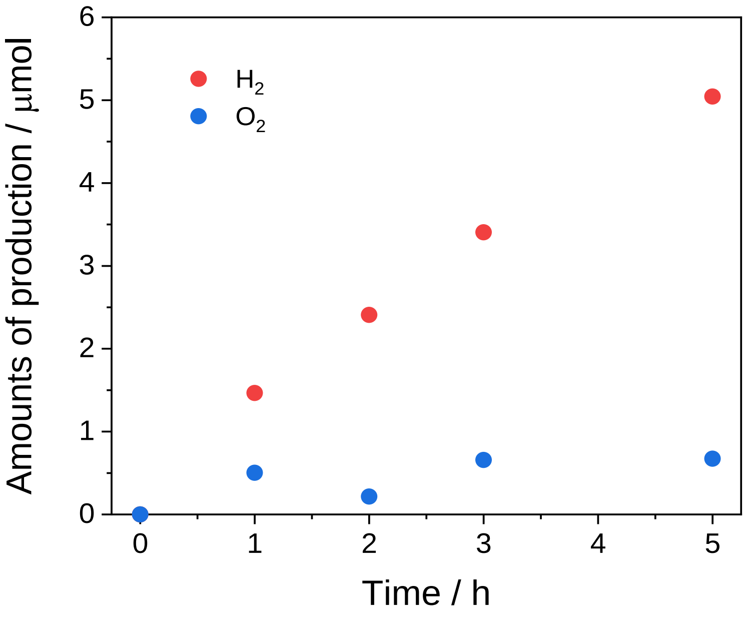

Figure S-12. Photocatalytic water splitting over **P10-Ir** (3 mg) in water (120 mL, pH 10.04, pH was adjusted by KOH) in gas-closed circulation system. light source: Xe light source (PerkinElmer CERMAX PE300BF 300 W Xe light source) with a 420 nm cut-off filter in a top-irradiation cell with a Pyrex window, irradiation area: 33 cm^2.

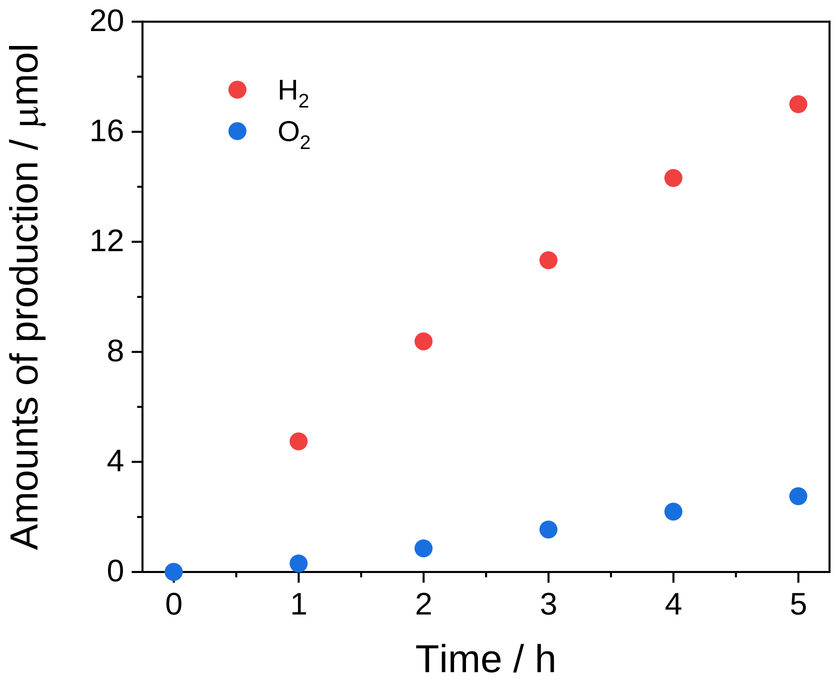

Figure S-13. Photocatalytic water splitting over **P10-10%Ir** (5 mg) in distilled water (120 mL) in gas-closed circulation system. light source: Xe light source (PerkinElmer CERMAX PE300BF 300 W Xe light source) with 420 nm cut-off filter in a top-irradiation cell with a Pyrex window, irradiation area: 33 cm^2.

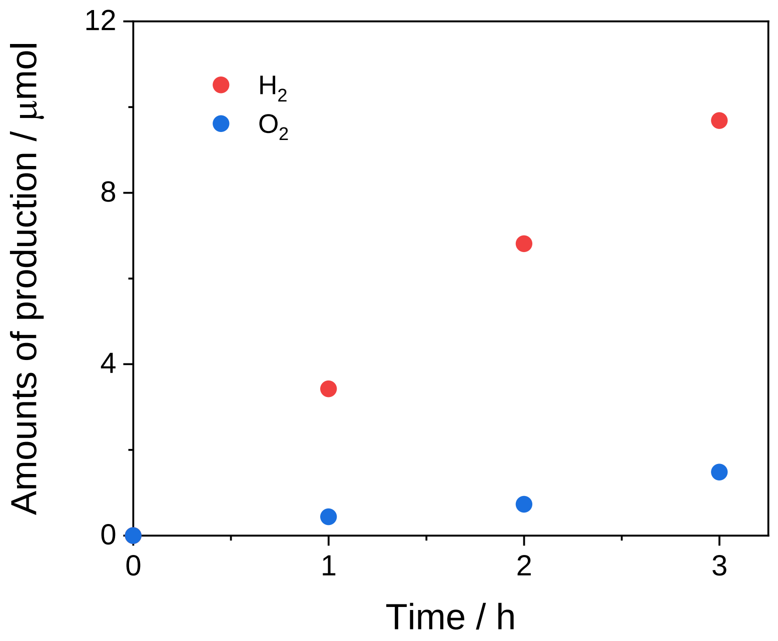

Figure S-14. Photocatalytic water splitting over **P10-2%Ir** (5 mg) in distilled water (120 mL) in gas-closed circulation system. light source: Xe light source (PerkinElmer CERMAX PE300BF 300 W Xe light source) with a 420 nm cut-off filter in a top-irradiation cell with a Pyrex window, irradiation area: 33 cm^2.

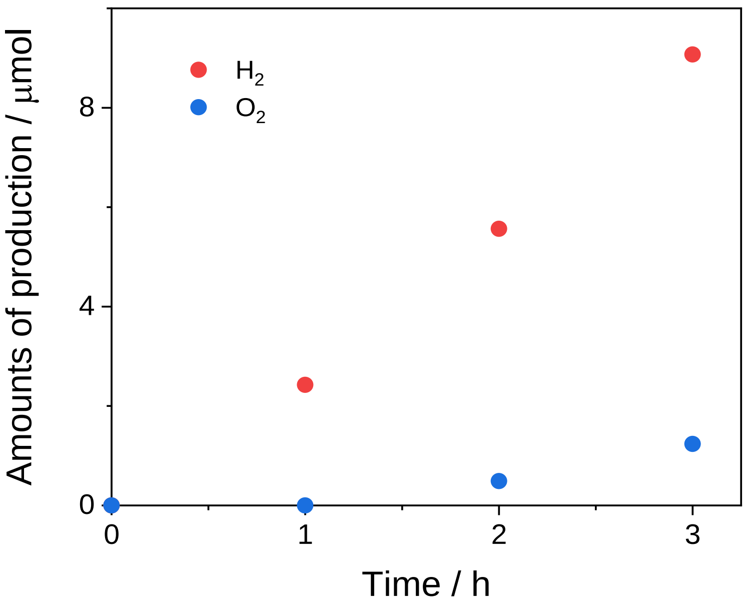

Figure S-15. Photocatalytic water splitting over **P10-0.5%Ir** (5 mg) in distilled water (120 mL) in gas-closed circulation system. light source: Xe light source (PerkinElmer CERMAX PE300BF 300 W Xe light source) with a 420 nm cut-off filter in a top-irradiation cell with a Pyrex window, irradiation area: 33 cm^2.

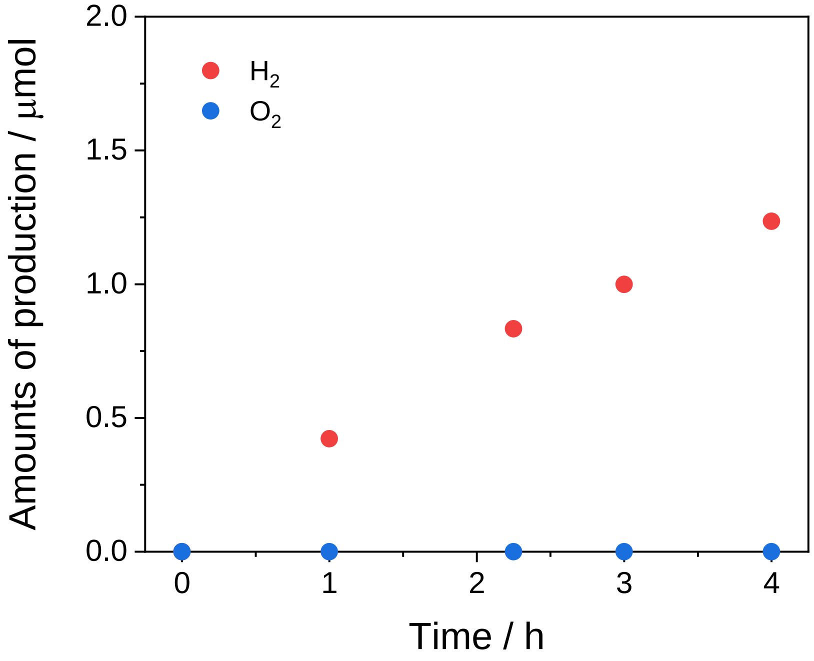

Figure S-16. Photocatalytic water splitting over **P10-Ru** (1 mg) in distilled water (120 mL) in gas-closed circulation system. light source: Xe light source (PerkinElmer CERMAX PE300BF 300 W Xe light source) with a 420 nm cut-off filter in a top-irradiation cell with a Pyrex window, irradiation area: 33 cm^2.

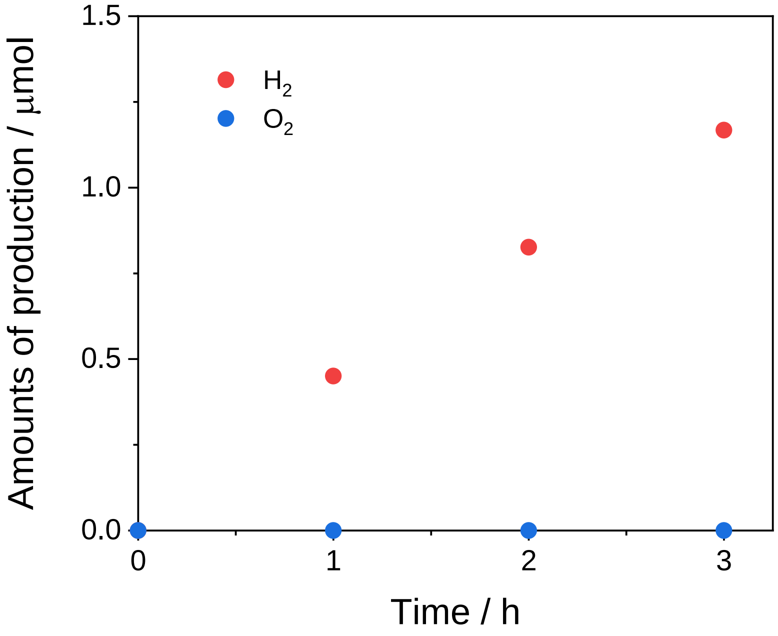

Figure S-17. Photocatalytic water splitting over **P10** (5 mg) in distilled water (120 mL) in gas-closed circulation system. light source: Xe light source (PerkinElmer CERMAX PE300BF 300 W Xe light source) with a 420 nm cut-off filter in a top-irradiation cell with a Pyrex window, irradiation area: 33 cm^2.

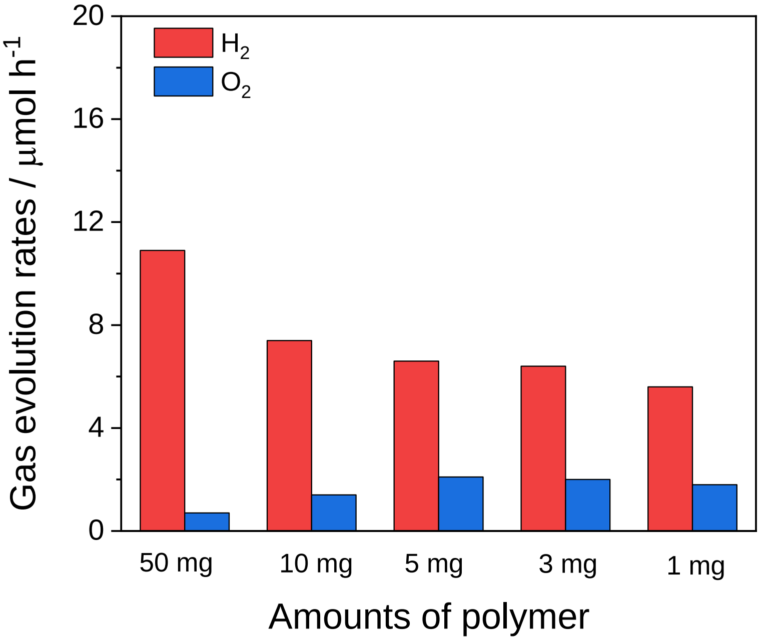

Figure S-18. Dependence of gas evolution rates on different amount of **P10-Ir** under visible light illumination (PerkinElmer CERMAX PE300BF 300 W Xe light source, $\lambda > 420$ nm) in a top-irradiation cell with a Pyrex window, using the data shown in Figs. S-23 to S-26 above, and Fig. 1d in main text.

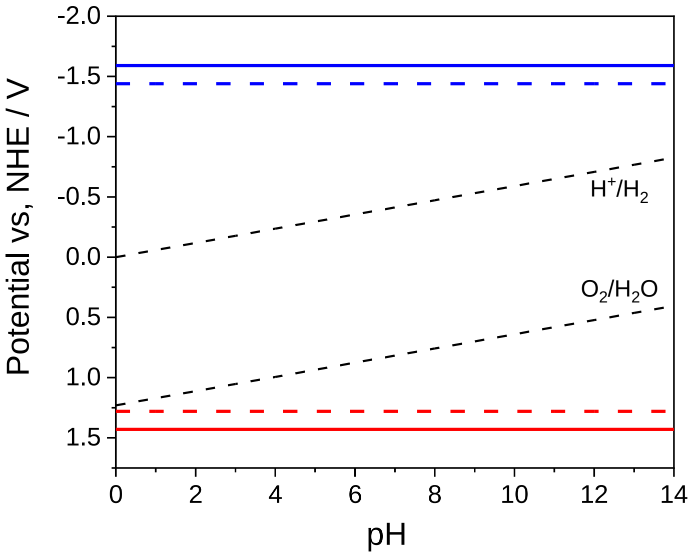

Figure S-19. Predicted ionization potential (IP, red solid line) and electron affinity (EA, blue solid line) of P10 with the exciton electron affinity (EA*, red dashed line) and ionization potential (IP*, blue dashed line), in comparison with the potentials for proton reduction (H_2/H^+), water oxidation (O_2/H_2O), in water for different pH values (underlying data taken from reference[1]).

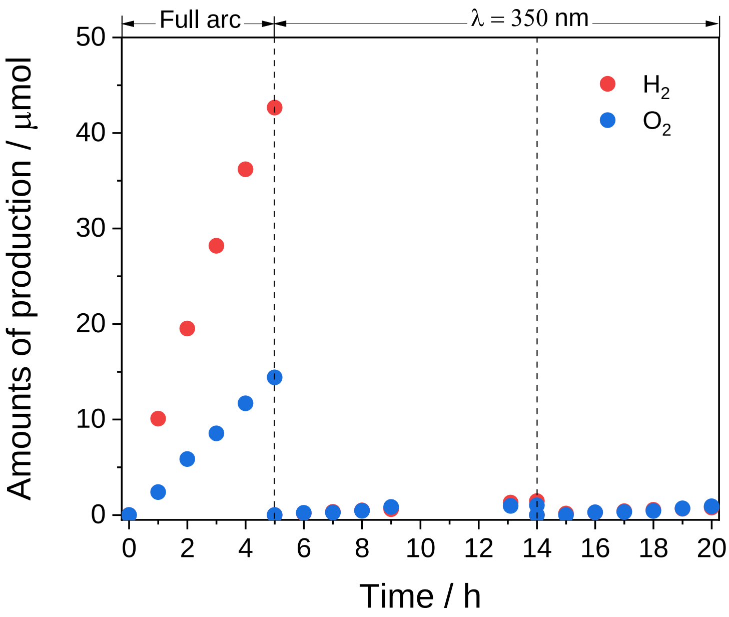

Figure S-20. Photocatalytic water splitting over **P10-Ir** (1 mg) in distilled water (120 mL) in gas-closed circulation system. light source: Xe light source (PerkinElmer CERMAX PE300BF 300 W Xe light source) with full arc illumination in first 5 hours, then with a 350 nm band pass filter in a top-irradiation cell with a Pyrex window, irradiation area: 33 cm^2. The AQY for hydrogen production was estimated to be 0.063% at 350 nm based on Equation 7. Note: The water splitting reaction yielded non-stoichiometric hydrogen and oxygen production.

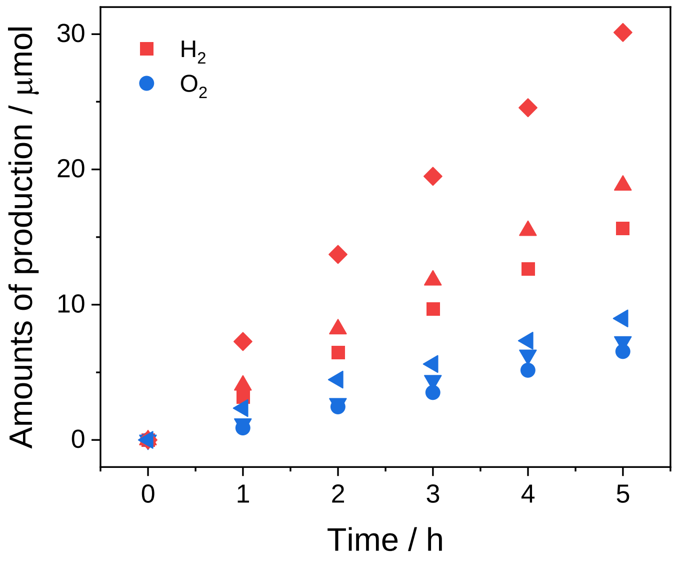

Figure S-21. Photocatalytic water splitting of **P10** (triangles, squares, and circles are using polymer photocatalyst from separate batches, red symbols relate to hydrogen production while blue symbols relate to oxygen production) in distilled water (120 mL) in gas-closed circulation system. Light source: Xe light source (PerkinElmer CERMAX PE300BF 300 W Xe, full arc) in a top-irradiation cell with a Pyrex window, irradiation area: 33 cm^2. Activities were measured after an initial stabilization period.

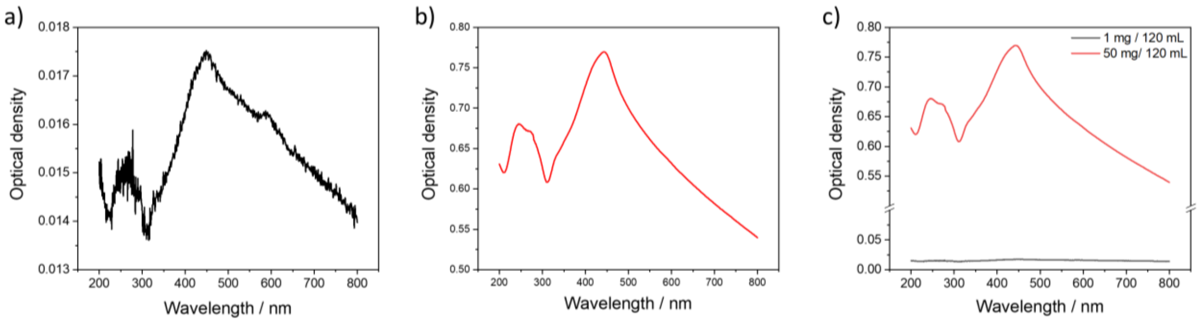

Figure S-22. UV-Vis absorption spectra of **P10-Ir** measured as suspensions in water with different concentrations: a) 8.3 mg L^{-1} (1 mg in 120 mL water), b) 0.4 mg L^{-1} (50 mg in 120 mL water), and c) comparison of 1 mg in 120 mL water and 50 mg in 120 mL water. All samples were measured after ultrasonication in a quartz cuvette with a 2 mm pathlength.

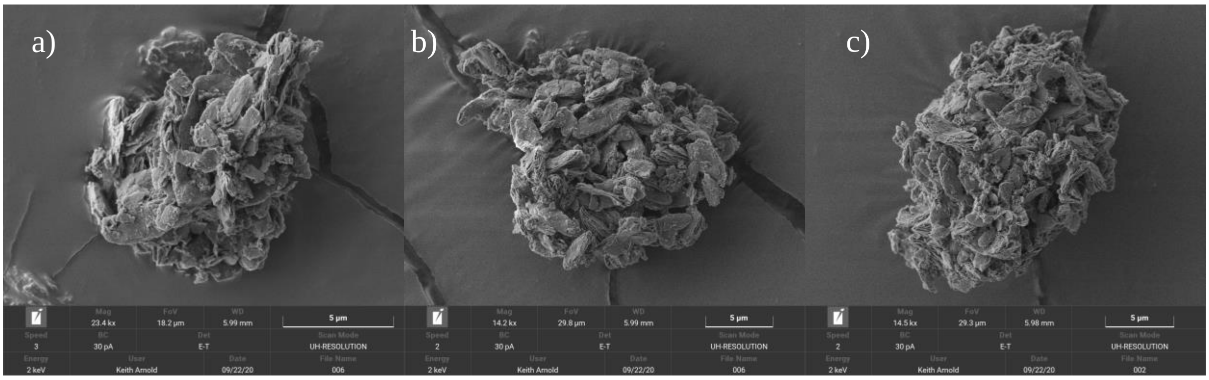

Figure S-23. SEM images of a) **P10,** b) **P10-Ir and** c) **P10-IrO_2**.

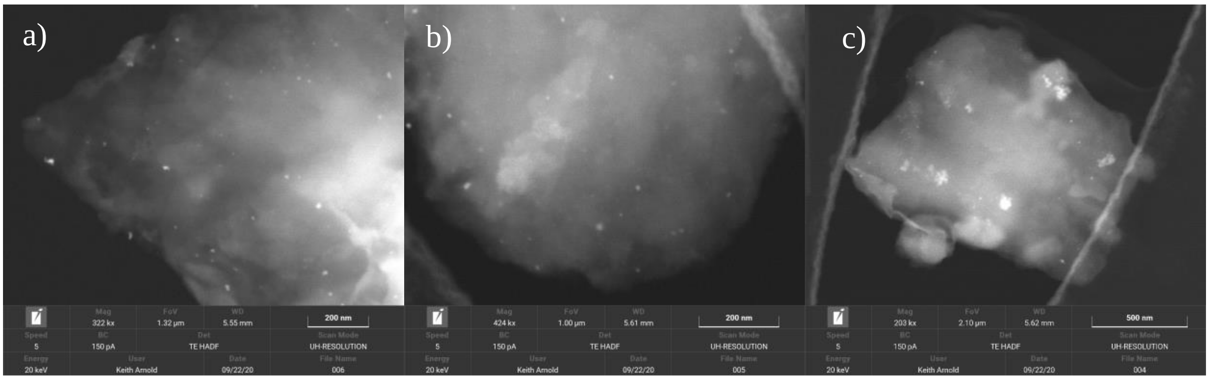

Figure S-24. STEM mode images of a) **P10,** b) **P10-Ir and** c) **P10-IrO$_2$**.

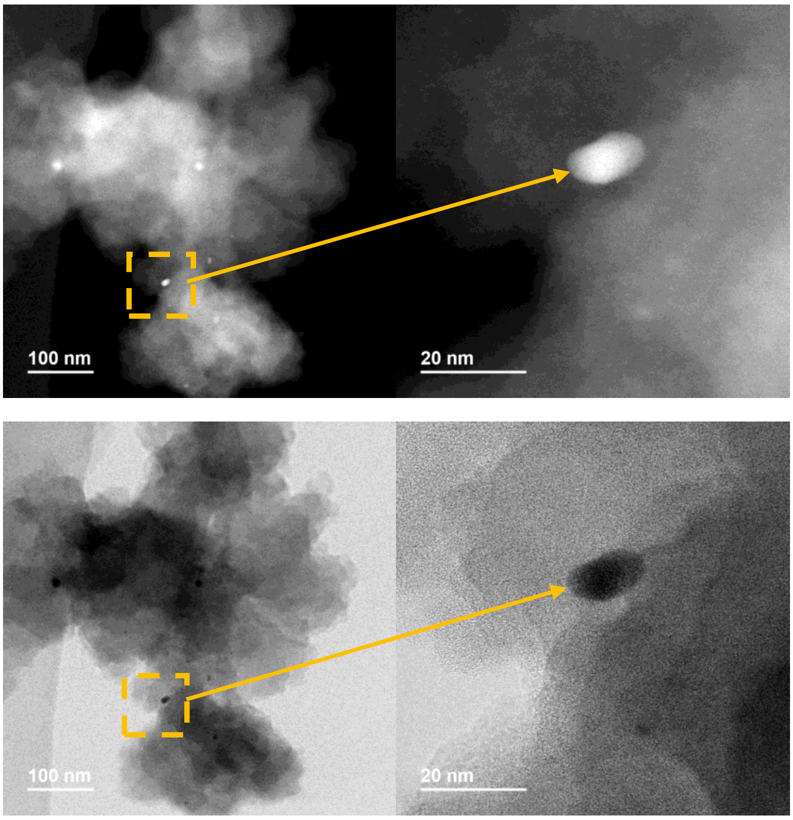

Figure S-25. STEM images of **P10** in HAADF (top) and BF (bottom) mode. The bright spot in the highlighted area in HAADF and the dark spot in BF are palladium particles originating from the synthesis.

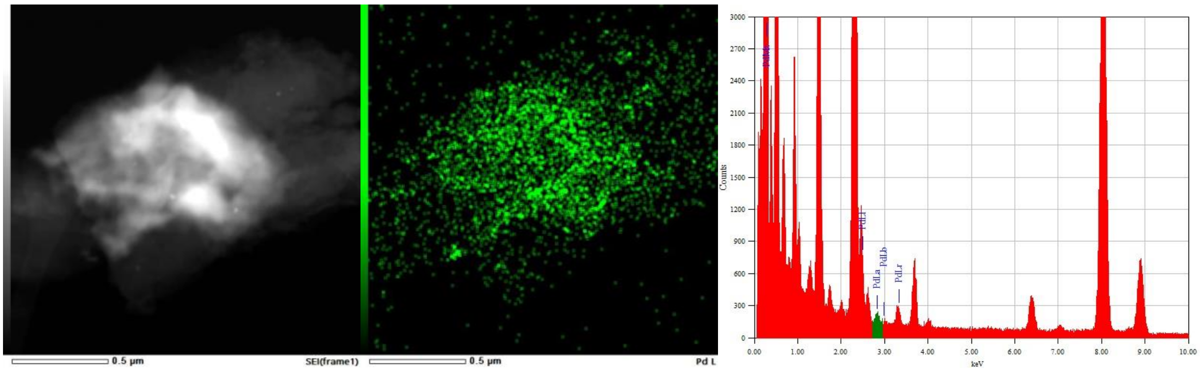

Figure S-26. STEM of **P10** (left), EDX palladium (L Shell) mapping of the same area (middle), and the EDX spectrum of P10 (right).

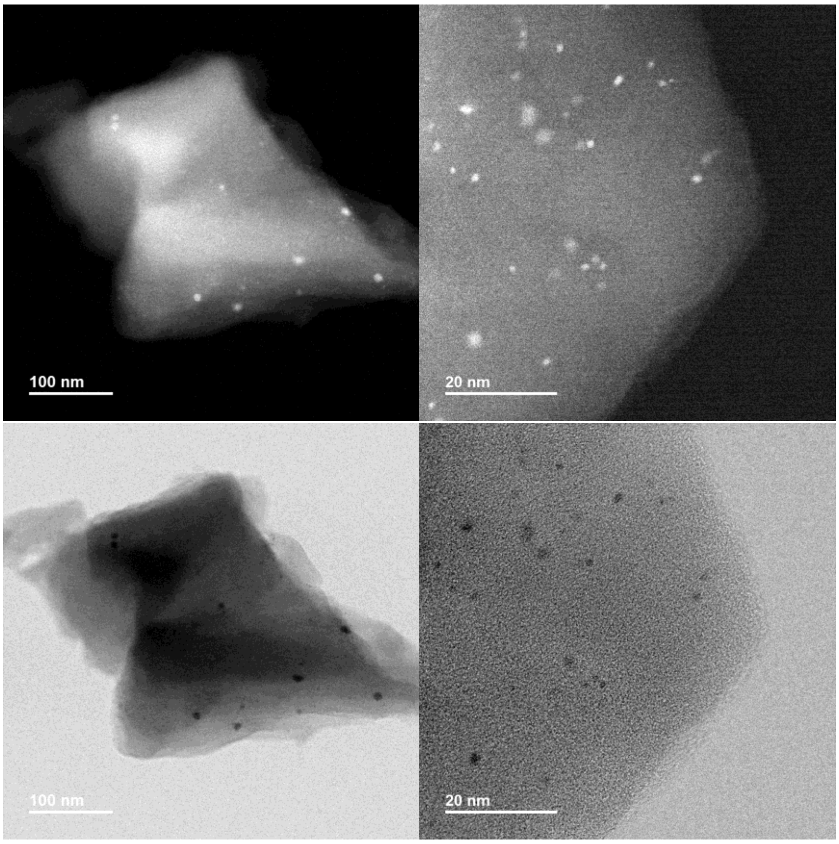

Figure S-27. STEM images of **P10-Ir** in HAADF (top) and BF (bottom) mode.

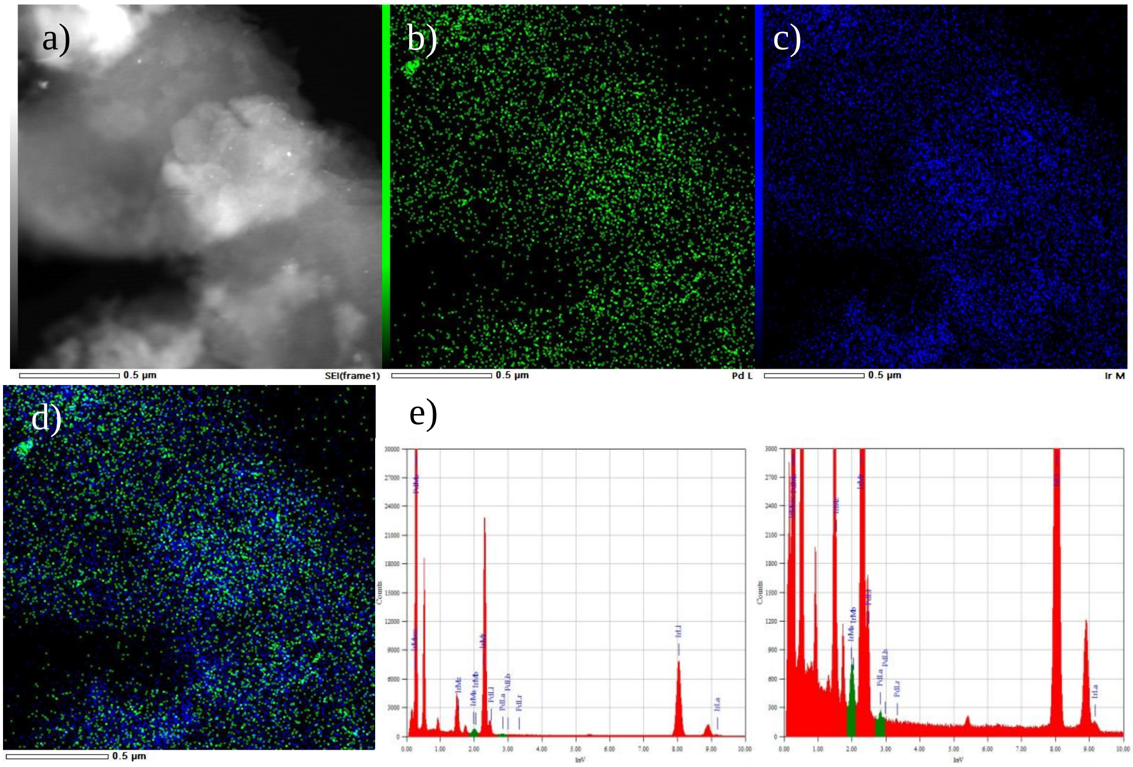

Figure S-28. a) STEM of **P10-Ir** and EDX b) palladium (L shell), c) iridium (M shell), d) palladium and iridium mapping of the same area, e) EDX spectrum and scaled spectrum of the Pd and Ir peaks. The L-alpha Pd energy is 2.9 KeV and the M-alpha Ir is around 1.9 KeV.

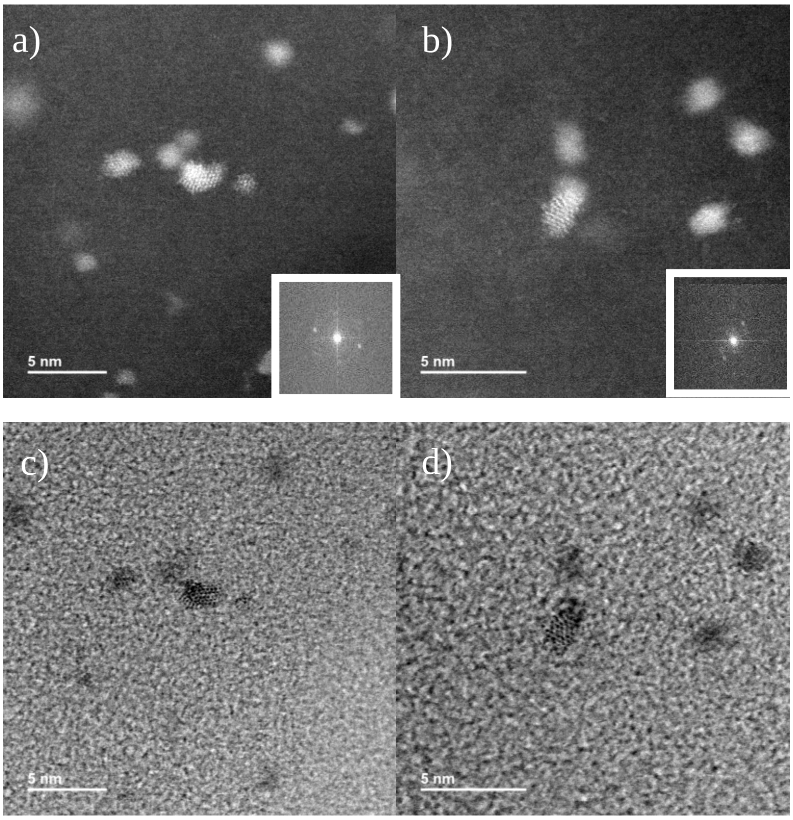

Figure S-29. HR-STEM images of **P10-Ir** in HAADF (a, b) and BF (c, d) mode. The fast Fourier transform (FFT) images inserted in a) and b) have a d-spacing of 0.203 nm (a), and 0.23 nm (b). The bright spot in the highlighted area in HAADF and the dark spot in BF are metallic iridium particles.

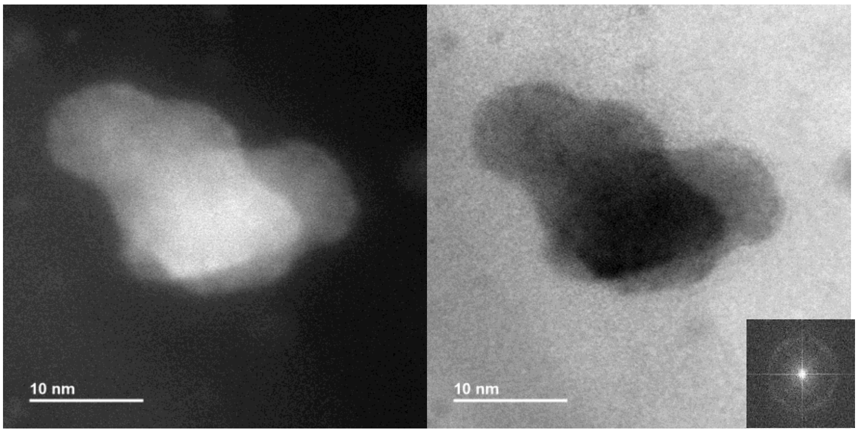

Figure S-30. STEM images of **P10-Ir** in HAADF (left) and BF (right) mode, the FFT image was inserted in STEM-BF with the d-spacing of 0.225 nm, the larger particle in this image is most likely IrO_2.

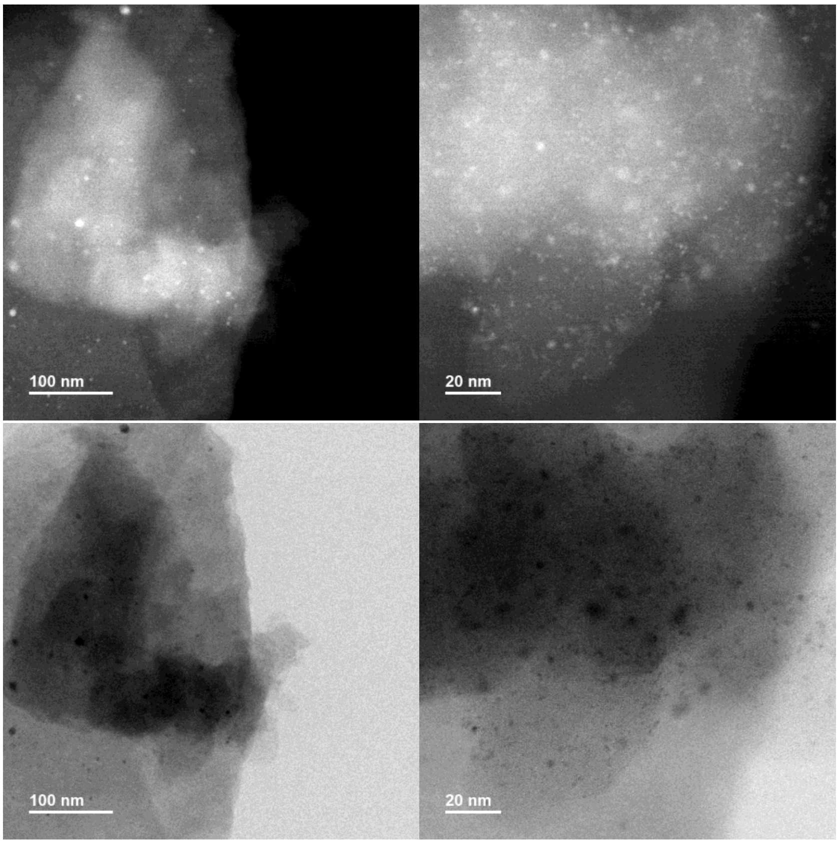

Figure S-31. STEM images of **P10-IrO_2** HAADF (top) and BF in mode (bottom).

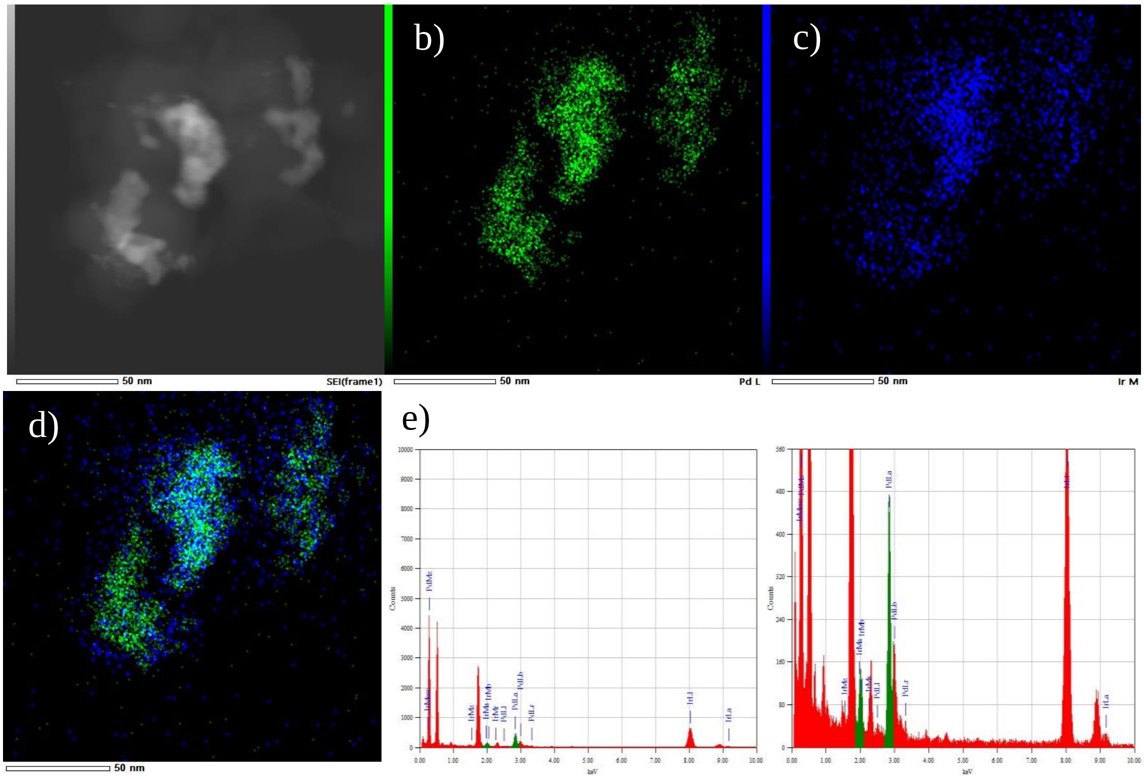

Figure S-32. a) STEM of **P10-IrO$_2$** and EDX b) palladium (L shell), c) iridium (M shell), d) palladium and iridium mapping of the same area, e) EDX spectrum and scaled spectrum of the Pd and Ir peaks. The L-alpha Pd energy is 2.9 KeV and the M-alpha Ir is around 1.9 KeV.

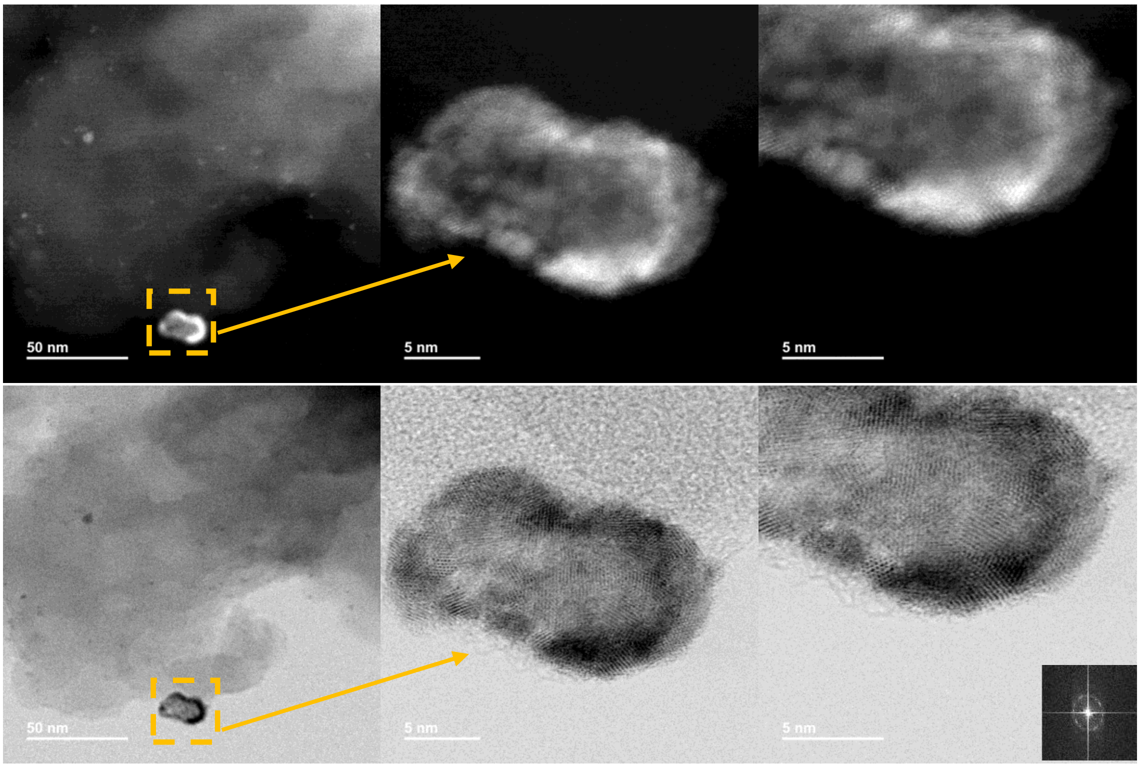

Figure S-33. HR-STEM images of **P10-IrO$_2$** HAADF (top) and BF in mode (bottom), the FFT image was inserted in STEM-BF with the d-spacing of 0.236 nm relating to IrO$_2$ (200) (JCPDS, 00-058-0335), the TEM image shows hollow IrO$_2$ structures on the polymer.

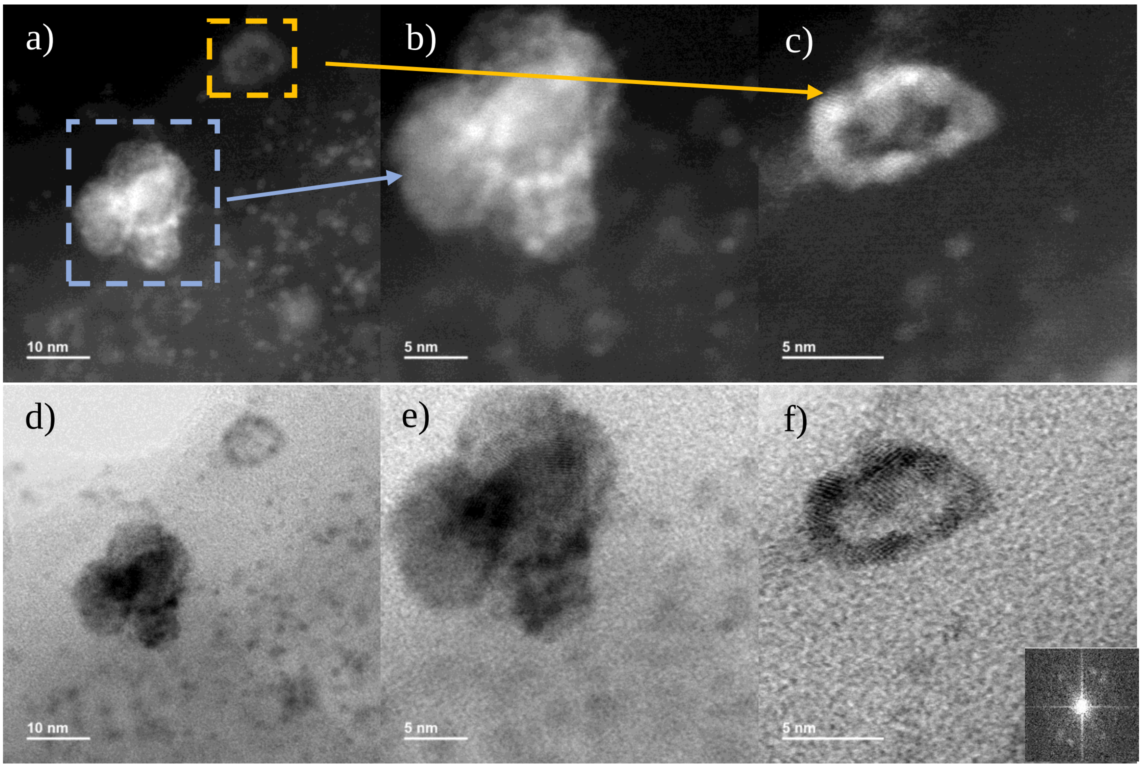

Figure S-34. HR-STEM images of **P10-IrO_2** HAADF (top) and BF in mode (bottom), b) and e) show particles containing both Pd and IrO_2, c) and f) show IrO_2, the FFT image was inserted in f) with the d-spacing of 0.225 nm.

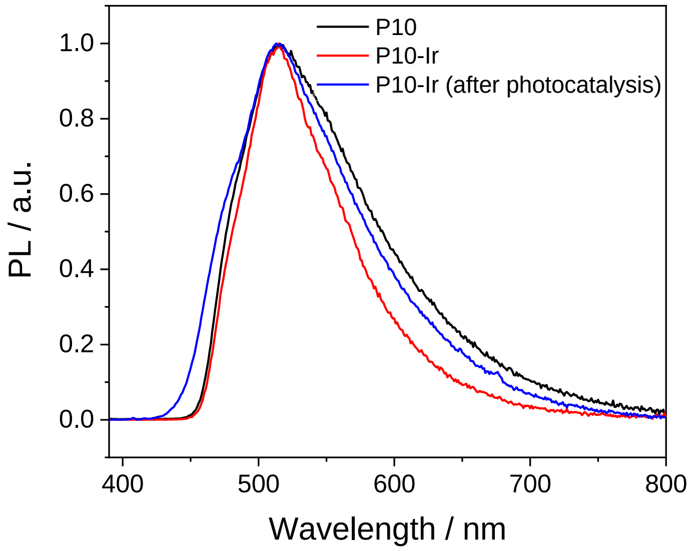

Figure S-35. Min-Max normalized emission spectra of **P10, P10-Ir**, and **P10-IrO_2** suspensions measured in water.

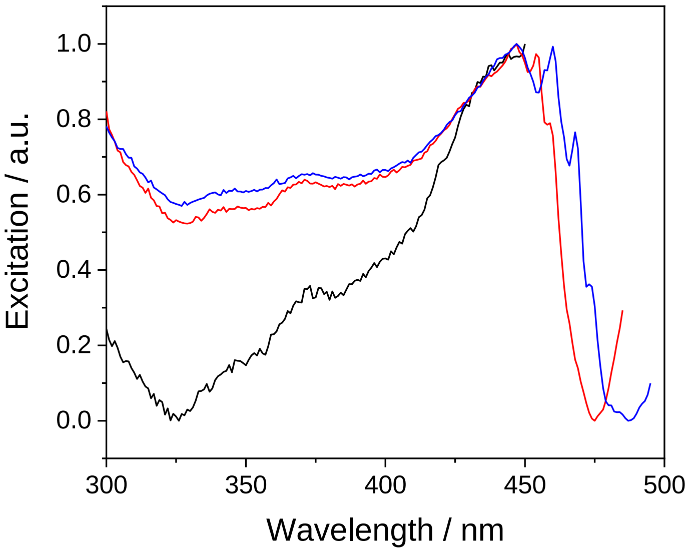

Figure S-36. Excitation spectra of **P10** suspensions measured in water for different emission peak values, black (470 nm), red (500 nm), and blue (515nm).

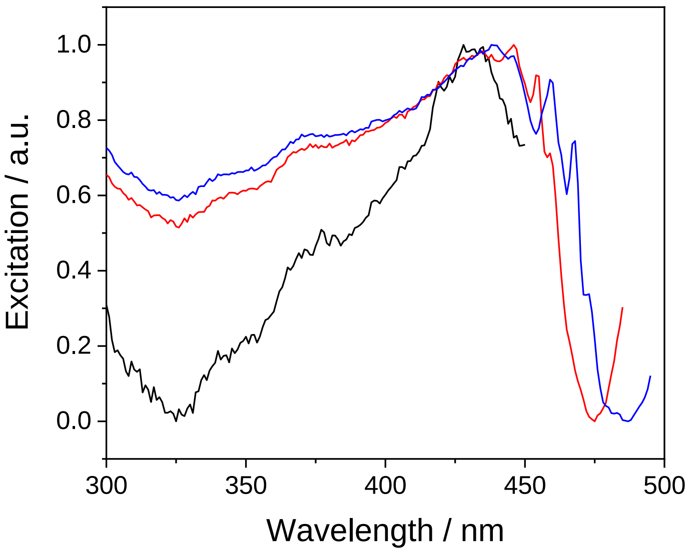

Figure S-37. Excitation spectra of **P10-Ir** suspensions measured in water for different emission peak values, black (470 nm), red (500 nm), and blue (515nm).

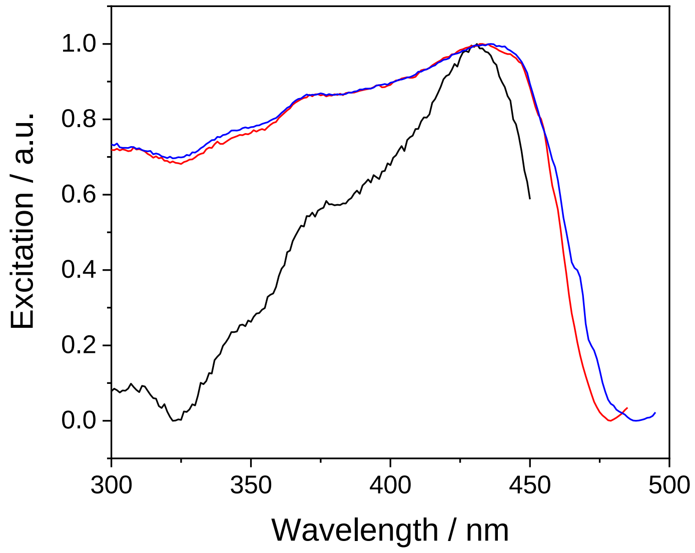

Figure S-38. Excitation spectra of **P10-IrO₂** suspensions measured in water for different emission peak values, black (470 nm), red (500 nm), and blue (515nm).

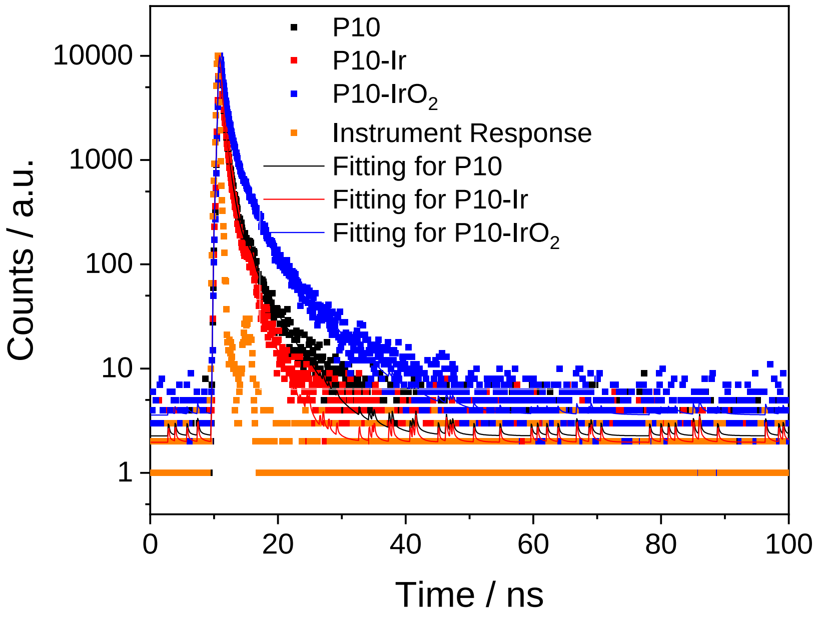

Figure S-39. Fluorescence life-time decays of **P10** (black dots), **P10-Ir** (red dots), and **P10-IrO₂** (blue dots) in water, instrument response (orange dots), and fitting for P10 (black line), P10-Ir (red line), and P10-Ir (after photocatalysis) (blue line).

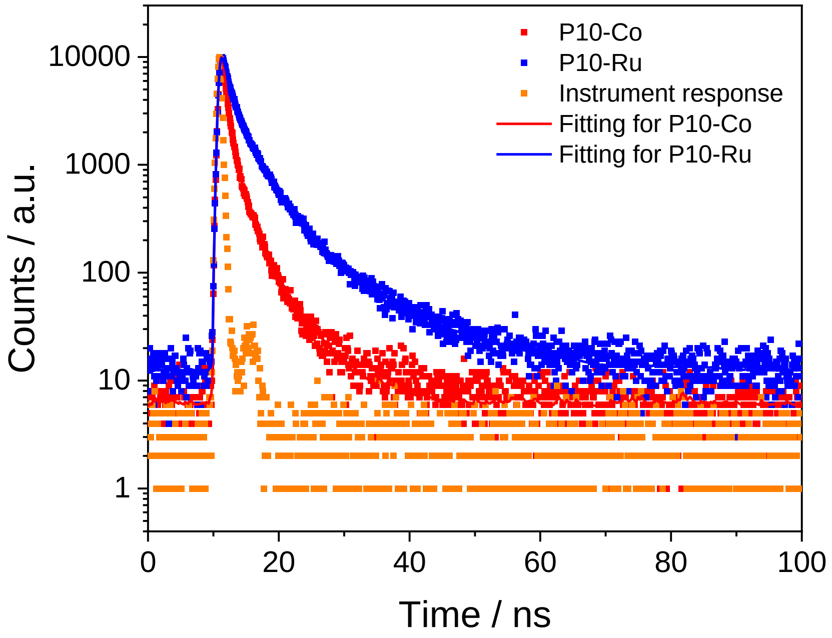

Figure S-40. Fluorescence life-time decays of **P10-Co** (red dots) and **P10-Ru** (blue dots) in water, instrument response (orange dots), and fitting for **P10-Co** (red line) and **P10-Ru** (blue line).

Table S-2. Estimated fluorescence lifetimes of **P10** suspensions in water from deconvolution fits.

**Polymer**	**τ_1 / ns**	**B_1 / %**	**τ_2 / ns**	**B_2 / %**	**τ_3 / ns**	**B_3 / %**	χ^2	**τ_{AVG} / ns**
**P10**	0.225	55.21	0.953	36.04	4.393	8.75	1.395	0.85
**P10-Ir**	0.158	47.38	0.685	43.22	2.616	9.40	1.284	0.62
**P10-IrO_2**	0.503	45.56	1.786	41.45	6.444	12.99	1.588	1.81
**P10-Co**	0.531	56.23	1.717	35.44	5.794	8.33	1.542	1.39
**P10-Ru**	1.116	38.10	3.671	49.40	11.589	12.50	1.057	3.69

[a] Fluorescence life-times for **polymers** in ethanol solution suspension obtained from fitting time-correlated single photon counting decays to a sum of three exponentials, which yield τ_1, τ_2, and τ_3 according to $\sum_{i=1}^{n}(A + B_i \exp(-t/\tau_i))$. τ_{AVG} is the weighted average lifetime calculated as $\sum_{i=1}^{n} B_i \tau_i$.

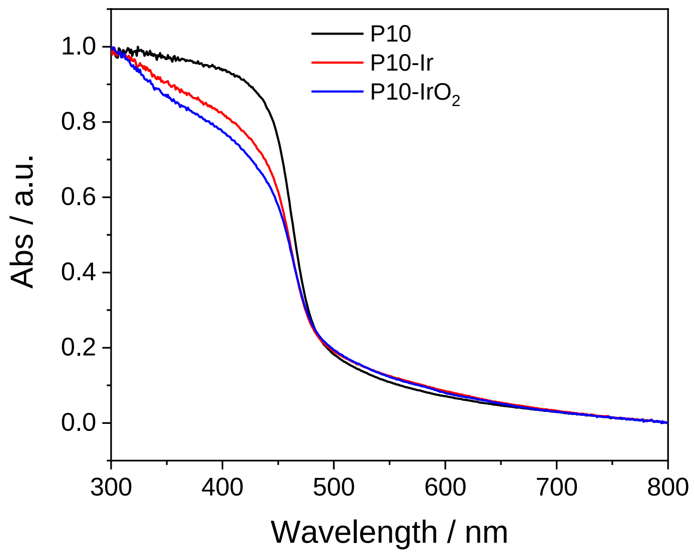

Figure S-41. Min-Max normalized UV-vis absorption spectra of **P10**, **P10-Ir**, and **P10-IrO$_2$** measured as powders in the solid-state.

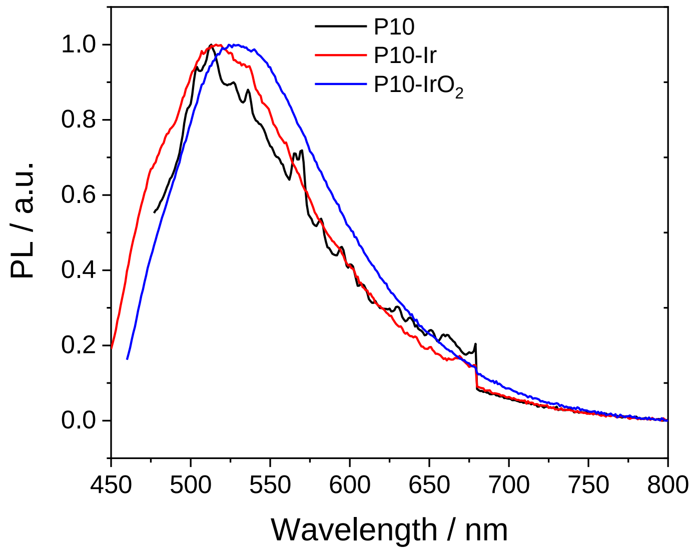

Figure S-42. Photoluminescence spectra (λ_{exc} = 350 nm) of **P10, P10-Ir,** and **P10-IrO$_2$** measured at room temperature as powders in the solid-state.

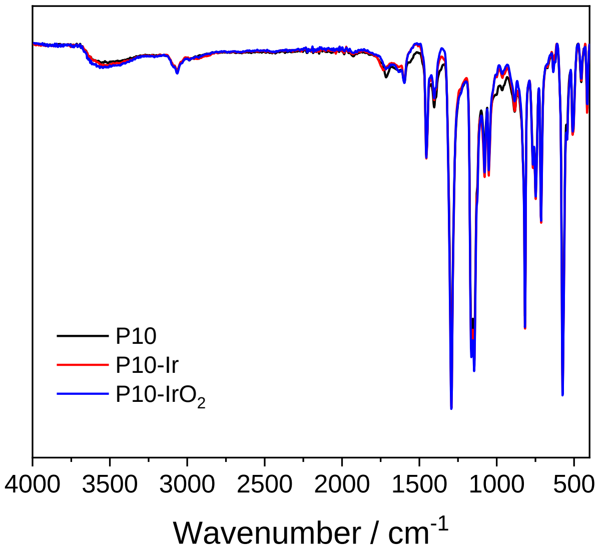

Figure S-43. Transmission FT-IR spectra of **P10** (black line)**, P10-Ir** (red line), and **P10-IrO$_2$** (blue line).

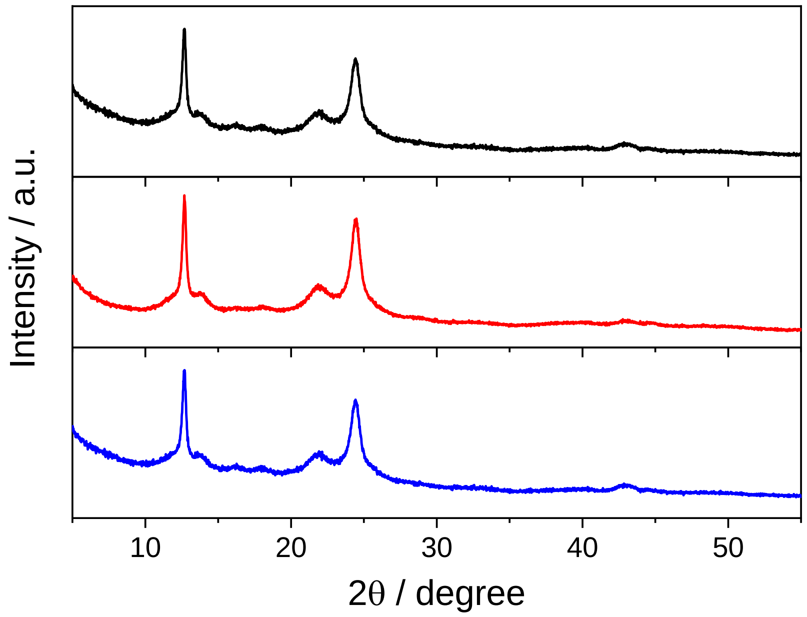

Figure S-44. pXRD patterns of **P10** (black line)**, P10-Ir** (red line), and **P10-IrO$_2$** (blue line).

Transient Absorption Spectroscopy experiments: Transient absorption spectra are recorded using a Harpia-TA spectrometer (Light Conversion) which is described elsewhere.[3] The 400 nm pump pulses with a power of 750 µW are generated using a Pharos-SP-10W (Light Conversion, FWHM ~140 fs, 10 kHz, 1030 nm) and a OPA (Orpheus, Light Conversion). The sample is pumped (excited) at an effective

rate of 5 kHz as a chopper within the Harpia discards 50% of pump pulses. A portion of the 1030 nm output is focused onto a sapphire crystral in the Harpia spectrometer to generate a white light probe at 10 kHz. The pump beam (ca. 0.6 mm diameter) and the probe beam (ca. 0.4 mm diameter) were overlapped on the sample position. Chirp dispersion is corrected manually within Carpetview (Light Conversion) and global fitting is carried out using the Carpetview software. Suspensions were prepared with P10 or P10-IrO_2 (post photocatalysis, 0.35 wt. % Ir loading) concentration of 0.24 g L^{-1} and ultrasonicated in water until the photocatalyst was dispersed (20 minutes), and the resultant suspensions were injected a quartz cuvette with a 2 mm pathlength, sealed with a rubber septa cap and degassed by Ar bubbling for 20 minutes. Samples were not stirred during ultrafast TAS measurements as they were found to be suitably stable during the TA experiment (ca. 30 minutes).

Global analysis: Initially global lifetime analysis (GLA) was carried out within the carpetview software. The principles of GLA and global target analysis (GTA) of TA data have been discussed in detail elsewhere.[3,4] The aim of GLA is to provide a way to visualise complex sets of time-resolved spectra by decomposing them into a small number of compartment populations and to examine their time dependence. Here we use a parallel evolution GLA approach where the TA data is fitted to a small number of compartments which are all initially populated and the population of each compartment is modelled by a single exponential decay function, which will be convoluted (⊗) by an instrument response function (IRF(t,λ)), Eq 1. The measured TA spectrum consists of the sum of the contributions from each pathway.

$$\Delta A\ (t,\lambda) = \sum_{j=1}^{n} x_j(\tau_j,\lambda)\, e^{-t/\tau_j} \otimes IRF(t,\lambda) \qquad \text{Equation S-1}$$

Singular value decomposition (SVD) analysis was carried out on both data sets, and this indicated that four compartments (labelled 0,1,2,3) were required in the GLA fitting of the P10 and P10-IrO_2 data. The complex nature of the system under study, which likely contains multiple excited state decay pathways means that although the data is well fitted to 4 main decay spectra these should not be considered to be spectra of individual photochemical species. Global analysis and the associated decay associated spectra (DAS) provide a way to represent the complex TA spectra of P10 into compartments with common decay lifetimes.

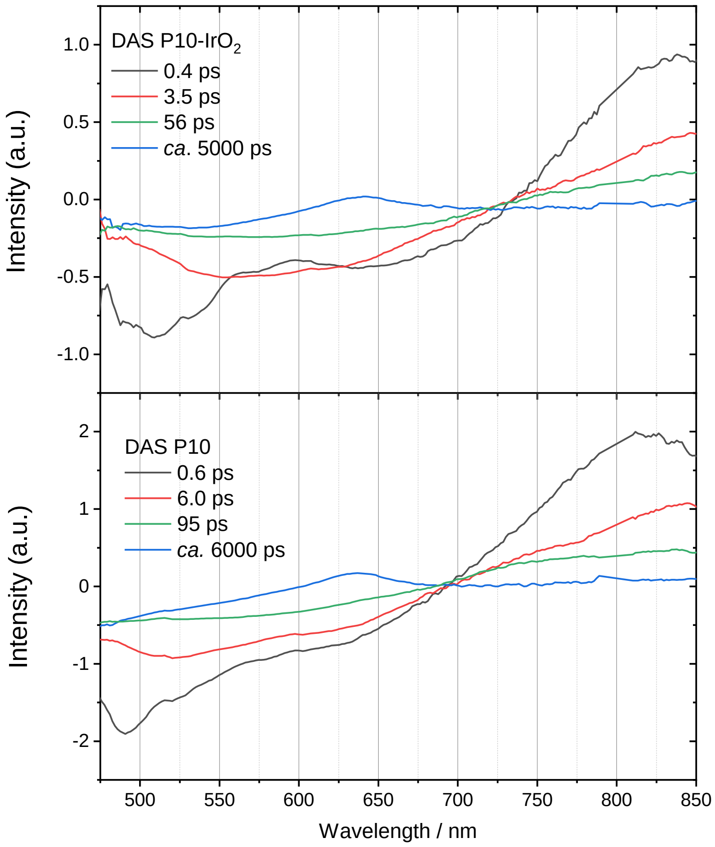

Figure S-45. DAS and associated lifetimes generated through a 4-compartment parallel pathway GLA of the TA data presented in the main text.

Figure S-45 shows the DAS and lifetime values for each component for both experiments. The *ca.* 0.6 ps DAS for P10 shows decay of the exicton state with a peak at 825 nm and a recovery of the bleach due to stimulated emission with peaks at 490 and 525 nm, in good agreement with the steady state PL spectrum. An additional broad feature centred around 630 nm in the 0.6 ps DAS for P10 suggests some electron or polaron pair formation on the ultrafast timescale in-line with our recent report of ultrafast polaron formation from hot excitonic states with P10.[5] The DAS for 6 ps is similar to the 0.6 ps except that the contribution from the higher energy emissive state is decreased, indicating that this is significantly shorter lived. Interestingly the 6 ps DAS at wavelengths > 700 nm appears to consist of two peaks around 750 and 830 nm. A small component with a 95 ps contains contributions from the photoinduced absorption decay at >750 nm, stimulated emission and the rise of the polaron band at 630 nm. Finally, the longest lived component (> 6000 ps) is dominated by a band at 630 nm which is due to long-lived electron polarons/polaron pairs, in agreement with the photocatalysis data that shows some H_2 evolution for P10 in water. Global analysis of the TA spectra recorded for P10-IrO_2 shows similar

behaviour however, there are three important differences. The lifetimes of compartments 0,1,2 are all decreased. The relative contribution from emission from the higher energy state (475 nm) in compartment 0,1 is decreased when compared to the contribution from the electron polaron state (~630 nm). Finally, the DAS of compartment 3 (*ca*. 5 ns (P10) and 6 ns (P10-IrO_2) shows a change in shape below 550 nm. A difference spectrum can be generated by subtracting the DAS associated with the longest-lived compartments of P10 and P10-IrO_2 (Figure S-46) which shows a broad absorption between 600-750 nm and an increase in absorption below 550 nm. The difference spectrum from the DAS in figure S-46 is in excellent agreement with that obtained through the GTA fitting presented in the main text supporting our assignment of a $P10^{(-)}$-$IrO_2^{(+)}$ state.

Overall GLA shows (i) that the broad band at >700 nm decays at different rates at different wavelengths and that it is likely consists of two excited states, with one potentially being the vibrationally hot state, on the basis of the changing shape of the DAS which show two peaks at different intensities and this is in-line with a recent transient vibrational spectroscopic study of P10.[5] (ii) The generation of the P10 polaron occurs on timescales that are common with the decay of the excited states and the excited states can also decay by relaxation to the ground state. (iii) The DAS of compartment 3 show clear differences between the two samples, which is in-line with a $P10^{(-)}$-$IrO_2^{(+)}$ state. (iv) The earliest DAS of P10-IrO_2 show a greater contribution from the increase in absorption of the polaron state indicating that it can be formed at a greater rate. Overall the rate of decay of DAS containing contributions which could be assigned to excitonic species is greater with P10-IrO_2.

GTA provides a way to generate species associated spectra (SAS) and on the basis of the GLA we are able to put forward the kinetic model in the main text for GTA. In this, two excited (excitonic) states are generated sequentially assigned to the initially formed vibrationally hot state and the thermalized excitonic state, both of which can lead to generation of the charges-separated state, or they can decay back to the ground state.

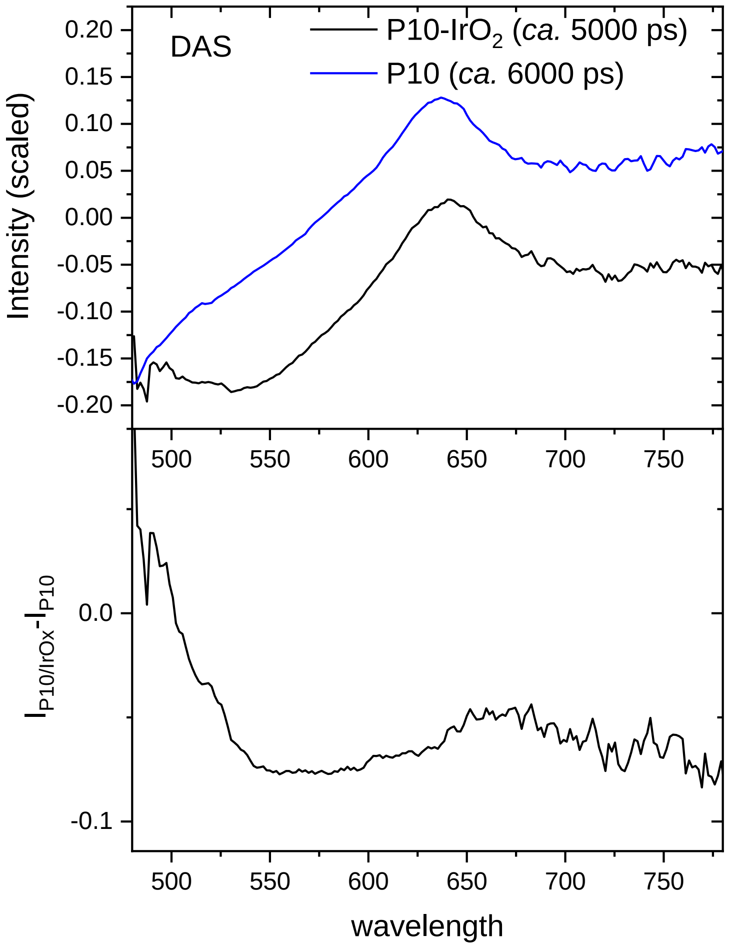

Figure S-46. Comparison of the DAS of the longest-lived compartment (top) and difference of DAS spectrum achieved by subtracting the **P10** DAS (black) from the **P10-IrO₂** DAS (blue) in the top panel (bottom).

The turnover number for polymer photocatalyst was calculated using the following equation:[6]

$$\mathrm{TON} = \frac{\text{Number of reacted holes}}{\text{Moles of catalyst}} \qquad \text{Equation S-2}$$

$$\mathrm{TON} = \frac{\text{Moles of oxygen produced} \times 4}{\text{Molecular weight of the repeat unit of P10}} \qquad \text{Equation S-3}$$

The solar energy conversion efficiency (STH) was calculated using the following equation (2):[7]

$$\eta_{STH}(\%) = \frac{[\text{Output energy as } \mathrm{H_2}(\mathrm{J})]}{[\text{Energy density of incident light } (\mathrm{J\ cm^{-2}})] \times [\text{Irradiated area } (\mathrm{cm^{2}})]} \times 100 \qquad \text{Equation S-4}$$

$$\eta_{STH}(\%) = \frac{[\Delta G^{0}(\mathrm{H_2O})\ (\mathrm{J\ mol^{-1}})] \times [\text{Rate of } \mathrm{H_2} \text{ evolution } (\mathrm{mol\ h^{-1}})]}{3600\ \mathrm{s} \times [\text{Solar energy (AM1.5) } (\mathrm{W\ cm^{-2}})] \times [\text{Irradiated area } (\mathrm{cm^{2}})]} \times 100 \qquad \text{Equation S-5}$$

$$\eta_{STH}(\%) = \frac{[237\ \mathrm{kJ\ mol^{-1}}] \times [\text{Rate of } \mathrm{H_2} \text{ evolution } (\mathrm{\mu mol\ h^{-1}})]}{3600\ \mathrm{s} \times [100\ \mathrm{mW\ cm^{-2}}] \times [25\ \mathrm{cm^{2}}]} \times 100 \qquad \text{Equation S-6}$$

The apparent quantum yields (AQY) was calculated using the follow equation:[8]

$$\eta_{AQY}(\%) = \frac{[(\text{The number of evolved H2 molecules) x 2}]}{[\text{The number of incident photons}]} \times 100 \qquad \text{Equation S-7}$$

Reference

[1] M. Sachs, R. S. Sprick, D. Pearce, S. A. J. Hillman, A. Monti, A. A. Y. Guilbert, N. J. Brownbill, S. Dimitrov, X. Shi, F. Blanc, M. A. Zwijnenburg, J. Nelson, J. R. Durrant, A. I. Cooper, *Nat. Commun.* **2018**, *9*, 1–11.

[2] M. Sachs, H. Cha, J. Kosco, C. M. Aitchison, L. Francàs, S. Corby, C. L. Chiang, A. A. Wilson, R. Godin, A. Fahey-Williams, A. I. Cooper, R. S. Sprick, I. McCulloch, J. R. Durrant, *J. Am. Chem. Soc.* **2020**, *142*, 14574–14587.

[3] M. Forster, D. W. F. Cheung, A. M. Gardner, A. J. Cowan, *J. Chem. Phys.* **2020**, *153*, 150901.

[4] I. H. M. Van Stokkum, D. S. Larsen, R. Van Grondelle, *Biochim. Biophys. Acta - Bioenerg.* **2004**, *1657*, 82–104.

[5] V. L. Piercy, K. H. Saeed, A. W. Prentice, G. Neri, C. Li, A. M. Gardner, Y. Bai, R. S. Sprick, I. V. Sazanovich, A. I. Cooper, M. J. Rosseinsky, M. A. Zwijnenburg, A. J. Cowan, *J. Phys. Chem. Lett.* **2021**, *12*, 10899–10905.

[6] Y. Sasaki, A. Iwase, H. Kato, A. Kudo, *J. Catal.* **2008**, *259*, 133–137.

[7] Q. Jia, A. Iwase, A. Kudo, *Chem. Sci.* **2014**, *5*, 1513–1519.

[8] K. Watanabe, Y. Iikubo, Y. Yamaguchi, A. Kudo, *Chem. Commun.* **2021**, *57*, 323–326.
